# From the Field to the Lab: Physiological and Behavioural Consequences of Environmental Salinity in a Coastal Frog

**DOI:** 10.3389/fphys.2022.919165

**Published:** 2022-06-02

**Authors:** Léa Lorrain-Soligon, Coraline Bichet, Frédéric Robin, François Brischoux

**Affiliations:** ^1^ Centre d’Etudes Biologiques de Chizé, CEBC UMR 7372 CNRS–La Rochelle Université, Villiers en Bois, France; ^2^ LPO France, Fonderies Royales, Rochefort, France; ^3^ Réserve Naturelle de Moëze-Oléron, LPO, Plaisance, Saint-Froult, France

**Keywords:** coastal ecosystems, immunological changes, locomotor performances, osmolality, Pelophylax sp., salinization

## Abstract

Environmental salinization is recognized as a global threat affecting biodiversity, particularly in coastal ecosystems (affected by sea level rise and increased frequency and severity of storms), and the consequent osmoregulatory challenges can negatively affect wildlife. In order to assess whether coastal species can respond to changes in environmental salinity, it remains essential to investigate the consequences of exposure to salinity in an environmentally-relevant context. In this study, we assessed the consequences of exposure to environmental salinity in coastal frogs (*Pelophylax* sp., N = 156) both in the field and experimentally, using a comprehensive combination of markers of physiology, behaviour and ecology. Exposure to salinity in the field negatively affected physiological parameters (osmolality, monocytes and eosinophils counts), as well as body condition and locomotor performance, and influenced size- and sex-specific habitat selection. Further, we demonstrated in a controlled experiment that short-term exposure to salinity strongly affected physiological parameters (salt influxes, water effluxes, immunity-related stress markers) and locomotor performance. Most of these effects were transient (water and salt fluxes, locomotor performance) once optimal conditions resumed (i.e., freshwater). Taken together, our results highlight the need to investigate whether exposure to environmental salinity can ultimately affect individual fitness and population persistence across taxa.

## 1 Introduction

Current global changes are causing numerous modifications to both abiotic and biotic conditions, which threatens biodiversity (Vitousek et al., 1997; Pievani, 2014). Although research has highlighted the detrimental consequences of some relatively conspicuous and mediatized components of global changes (e.g., global warming, Walther et al., 2002; land-use changes and habitat loss, Jantz et al., 2015; invasive species, Early et al., 2016), other—presumably less obvious—environmental parameters have been proportionally less studied. A typical example of such a scarcely studied impact of global change is environmental salinization (Cunillera-Montcusí et al., 2022). Yet, environmental salinization is recognized as a global threat currently affecting oceanic, freshwater and terrestrial ecosystems worldwide (Herbert et al., 2015). For instance, oceanic salinity has increased in areas where evaporation exceeds precipitation (Helm et al., 2010), but also in colder areas due to global climate changes (e.g., Antarctic, Meredith and King, 2005), potentially inducing major shifts in species distributions (Brischoux et al., 2021). A large proportion of Earth’s freshwater wetlands have already been affected by salinization (Cañedo-Argüelles, 2020; Cunillera-Montcusí et al., 2022; Hébert et al., 2022), inducing large shifts in wetland communities and their associated ecosystem functions (Herbert et al., 2015). Even terrestrial environments can be affected by this process (Daliakopoulos et al., 2016), which can deteriorate soil fertility in irrigated arable lands (Singh, 2019; Ondrasek and Rengel, 2021), constrain soil-plant-water transfers (Munns et al., 2020), and affect microbial activity and carbon balance (Gavrichkova et al., 2020).

Coastal habitats—recognized as some of the most diverse and productive habitats on Earth (Hobohm et al., 2021)—are particularly at risk of increasing salinization because of their position at the boundary between land and sea (McLean et al., 2001). Indeed, although coastal wetlands are frequently subjected to moderate salinity levels (i.e., sea spray deposition linked to landward winds, Benassai et al., 2005), they have become particularly exposed to salinization because of two processes that function at different temporal scales. First, predicted sea level rise should induce a progressive increase in environmental salinity of coastal wetlands (Morris et al., 2002), that will ultimately produce landward shifts of these habitats where it is possible (Di Nitto et al., 2014; Field et al., 2016). Second, predicted increases in the frequency and intensity of extreme weather events (i.e., storms and associated marine floods, Dettinger 2011) are expected to induce sudden, unpredictable and abrupt salinization (Delaune et al., 2021).

Salinization-induced osmoregulatory challenges are expected to alter ecological assemblages and exert severe constraints on wildlife (Cañedo-Argüelles et al., 2013; Herbert et al., 2015). Indeed, physiological adaptations are required to live in a specific range of salinity (Beadle, 1957; Kirschner, 1991; Hallows and Knauf, 1994), and coping with high salinity (exceeding the species tolerance range) can be challenging (Rivera-Ingraham and Lignot, 2017). Mechanisms involved in osmoregulation are metabolically costly and thus should be traded off against allocation to other functions, such as growth (Munns and Tester, 2008), reproduction (Morris et al., 2002; Herbert et al., 2015), and activity (Santos et al., 2007; Van Meter and Swan, 2014). Exposure to relatively high salinity can also induce more direct effects, such as malformations (Morris et al., 2002; Santos et al., 2007) or cell damage (Koleva et al., 2017). Most coastal species are subjected to relatively moderate levels of salinity in their day-to-day life (Greenberg et al., 2006), and living in such environments may provide a powerful selective context allowing these species to display adaptive responses to tolerate salinity (Purcell et al., 2008; Hopkins et al., 2016). In order to assess whether coastal species can adapt to the expected changes in environmental salinity, it remains essential to investigate the consequences of exposure to salinity in an environmentally-relevant context.

Amphibians are well-suited study models to investigate such a research question (Hopkins and Brodie, 2015). Although previously thought as mostly intolerant to elevated salinities, amphibians are relatively diverse and abundant in coastal wetlands (Hopkins and Brodie, 2015). In addition, they are strongly dependent on the availability of fresh water, and are particularly sensitive to salt because of their highly permeable skin (comparatively to other species), which is involved in water, gas and ion exchanges (Hillyard, 1999; Quaranta et al., 2009). In larvae, it has been observed that increased salinity affects whole body osmolality (Gomez-Mestre et al., 2004; Lukens and Wilcoxen, 2020; Tornabene et al., 2022), and negatively influences immune responses (Burraco and Gomez-Mestre 2016) and, in turn, increases the susceptibility of tadpoles to infections and diseases (Milotic et al., 2017; Hall et al., 2020). In addition, increased costs of osmoregulation have been shown to reduce physical performance (Gomez-Mestre et al., 2004; Kearney et al., 2016) and salinity has been shown to reduce activity, speed or movement (Squires et al., 2008; Denoël et al., 2010; Wood and Welch, 2015; Hall et al., 2017), affecting foraging efficiency (Chambers, 2011; Hall et al., 2017) and antipredator responses (Watkins, 1996; Squires et al., 2008; Johnson et al., 2015). Although adult life-stages should be affected by saline conditions (Hopkins and Brodie, 2015; Park and Do, 2020; Lorrain-Soligon et al., 2021), the physiological and behavioural consequences have been less studied than those in larvae. Given the influence of adult life-stages in population dynamics—and thus population persistence—in amphibians (Wells, 2007), such caveats can hamper our ability to thoroughly assess whether coastal amphibians can sustain the expected change in environmental salinity.

In this study, we adopted a comprehensive approach to assess the behavioural and physiological consequences of exposure to environmental salinity in adults of a coastal frog (*Pelophylax* sp., N = 156) originating from freshwater and brackish ponds in wetlands from the Atlantic coast of France. First, we used a field-based approach to assess the consequences of pond salinity on 1) physiology [osmolality (Gomez-Mestre et al., 2004), hemoglobin-binding protein (Wang et al., 2020), blood cell composition (Burraco and Gomez-Mestre 2016)], 2) locomotor performance [jumping distance, (Alexander et al., 2012; Kearney et al., 2016; Sanabria et al., 2018)] and 3) ecological responses [morphology and sex-ratio (Lorrain-Soligon et al., 2022b)]. On the same individuals, and for the same measures, we then used an experimental approach to precisely measure the consequences of short-term (48 h) exposure to different levels of salinity (0, 6, 9 or 12 g L^−1^) similar to those recorded in the field (min: 0 g L^−1^, max: 16 g L^−1^). This experiment was preceded and followed by acclimation in freshwater (0 g L^−1^) to assess whether individuals were able to recover from the effects of exposure to salinity.

First, we hypothesized that pond salinity should influence the distribution of individuals according to their body size (because water and ion fluxes should be linked to surface area to volume ratio, Gordon et al., 1961) and to their sex (because of the energetic cost of osmoregulation, Rivera-Ingraham and Lignot, 2017). Accordingly, we predicted that we should capture larger individuals and more males (as compared to females) in ponds with relatively high salinity. Second, we hypothesized that salinity should influence both physiological markers and performance (Gutiérrez, 2014). Accordingly, we predicted that, both in the field and under experimental conditions, high levels of salinity should increase plasmatic osmolality (Gomez-Mestre et al., 2004), increase hemoglobin-binding proteins concentration (as salinity induces damages to cells, Koleva et al., 2017), and increase the proportion of neutrophils compared to lymphocytes (Burraco and Gomez-Mestre 2016), but decrease jumping performance and activity (Alexander et al., 2012; Kearney et al., 2016; Sanabria et al., 2018). We predicted that these effects should dissipate when normal conditions (access to freshwater) are restored (Park and Do, 2020). Finally, we further hypothesized that individuals from brackish sites should be locally adapted (Hua and Pierce, 2013), and we predicted that individuals originating from high salinity ponds to be less impacted.

## 2 Methods

### 2.1 Study Site, Species and Field Procedures

#### 2.1.1 Study Site, Studied Species and Ethics Statement

The study was carried out on the “Réserve Naturelle Nationale de Moëze-Oléron” (45°53′33.36″N, 1°04′59.16″W), located in the Atlantic coast of France (Charente-Maritime). In the study area, *Pelophylax* sp. are composed of viable and fertile hybrids (Graf’s hybrid frog, *P. kl. grafi*) of the Marsh frog (*P. ridibundus*) and the Perez’s frog (*P. perezi*, Speybroek et al., 2018). These frogs are distinctly aquatic, and active both during the day and at night. In our study site, *Pelophylax* sp. can be found in ponds in which salinity ranges from 0.14 to 16.19 g L^−1^ (mean: 2.86 ± 2.17 g L^−1^).

This work was approved by the French authorities under permits R- 45GRETA-F1-10, 135-2020 DBEC and APAFIS#30169-2021022515546003 v3.

#### 2.1.2 Field Procedures

Individuals were captured at night, between 10 and 12 p.m. at 13 different ponds (each separated by less than 1.3 km from one another, see Supplementary Appendix A) from 29/03/2021 to 29/05/2021. Salinity (measured with a conductimeter YSI Professional Plus) at the capture sites ranged from 0.10 to 7.16 g L^−1^ (mean = 3.25 g L^−1^ ± 2.16 g L^−1^). On these sites, we captured a total of 156 individuals, 36 in freshwater ponds (<1 g L^−1^: range 0.10–0.76 g L^−1^, mean = 0.50 g L^−1^ ± 0.24 g L^−1^), and 120 in brackish ponds (>1 g L^−1^: range 1.46–7.16 g L^−1^, mean = 4.07 g L^−1^ ± 1.76 g L^−1^), as freshwater ponds were less common than brackish ones (see Supplementary Appendix B for details on capture dates, salinity of the pond, and sex of captured individuals). All captured individuals weighed more than 15 g, a threshold that allow sexing in our study population. Upon capture, individuals were brought to the laboratory, where they were weighed (with a portable electronic balance ±0.1 g) and measured for body size (snout-vent length: SVL) and hindlimb length (left leg) using a caliper (±0.1 mm). Sex was assessed based on the presence of secondary characteristics (Speybroek et al., 2018). We performed tests of jumping performance and evaluated activity scores (see below).

Finally, we collected blood samples (∼1% body mass, Diehl et al., 2001; Soulsbury et al., 2020) through cardiocentesis, using 1 ml syringes and heparinized 30G needles. These blood samples were performed within 2 h after capture (between 2 and 4 a.m.). Collected blood was placed in a 0.675 ml microcentrifuge tube and centrifuged (7 min at 2000 G), and plasma fractions were collected and stored at −18 °C in sealed microtubes until analyses.

### 2.2 Housing Conditions

Frogs were housed individually in transparent plastic boxes (14 × 16 × 9 cm) with a perforated cover to minimize evaporation while providing sufficient ventilation. Each box was filled with water (see details below) to allow permanent contact with water while allowing aerial respiration. Frogs were not fed during the experiment. They were kept in a room with natural photoperiod.

### 2.3 Experimental Design

Our experiment consisted of three successive stages:

1) Because frogs were captured in ponds with varying salinity (see above), boxes were first filled with freshwater (0.34 g L^−1^ salinity ± 0.007 SE) for 24 h to allow frogs to restore osmotic balance. This preparatory stage, hereafter termed “acclimation”, was intended to establish plasma normosmolality in all individuals. At the end of this stage, individuals were weighed, tests of jumping distance and activity scores were performed, and a blood sample was collected. Individuals were then randomly allocated to the experimental groups. Experimental groups were identical in term of mass, size (SVL) and sex distribution (all *p*-values > 0.751; see Supplementary Appendix C).
2) In the second stage (hereafter termed “Exposure”), we subjected for 48 h the frogs to one of four salinity levels: freshwater (0.35 g L^−1^ salinity ± 0.003 SE, N = 47 individuals, hereafter 0 g L^−1^ for simplicity) and three different brackish water treatments, namely 6 g L^−1^ (6.11 g L^−1^ salinity ± 0.01 SE, N = 46 individuals), 9 g L^−1^ (9.14 g L^−1^ ± 0.02 SE, N = 30 individuals) and 12 g L^−1^ (12.08 g L^−1^ salinity ± 0.040 SE, N = 33 individuals). Water was prepared by dissolving sea salt in tap water. Individuals were weighed every 24 h, while tests of jumping distance and activity scores were performed and a blood sample was collected at the end of the exposure to salinity treatments.
3) In the last stage (hereafter termed “Recovery”), each treatment water was replaced by freshwater (0.35 g L^−1^ salinity ± 0.009 SE) for 24 h in order to allow individuals to restore their osmotic balance. Jumping performance and activity scores were assessed at 12 and 24 h and a final body mass measure was collected at the end of this stage. Individuals were then released at the site of capture. We did not collect blood at the end of the recovery stage for ethical reasons (i.e., to limit both the number of punctures and the amount of blood withdrawn). We relied on an indirect assessment of osmotic balance at the end of the recovery stage by measuring salt effluxes (salinity of the water, see below). Relationships between salt fluxes and osmolality were assessed during the treatment stage of the experiment and verified that both parameters were linked (see below). Unfortunately, eight individuals that had been exposed to the 12 g L^−1^ treatment died, either during exposure or during recovery, and determinants of mortality (e.g., morphology) were analysed (see below).

### 2.4 Measurements

#### 2.4.1 Behavioural Parameters

##### 2.4.1.1 Activity Scores

We evaluated the condition of each individual using an activity score during daily manipulation (i.e., during weighing). These scores were set on a scale ranging from 1 to 5. To score activity, we referred to the following quantifications: 1) Weak individuals with low muscular strength (which is scored by the experimenter holding the frog and evaluating its posture and its reaction to manipulation) and no tendency to engage in escape behaviour, no or very few movements (individuals were almost motionless). These individuals did not jump if not stimulated. 2) Weak individuals with low muscular strength, and no tendency to engage in escape behaviour, few movements. These individuals responded weakly to manipulation but did not jump if not stimulated. 3) Individuals moved, had low muscular strength, tried to evade manipulation, jumped even if not stimulated. These individuals did not move energetically and stopped jumping after one or two jumps if not stimulated. 4) Individuals moved energetically, tried to evade manipulation, had perceptible muscular strength, jumped even if not stimulated, but did stop jumping after five or six jump if not stimulated. 5) Individuals moved energetically, tried to evade manipulation, had perceptible muscular strength, jumped even if not stimulated, and did not stop jumping and moving during whole duration of the experiment. For consistency, all activity score assessments were performed by the same person (LLS).

##### 2.4.1.2 Jumping Distances

Jumping performance was assessed by measuring the distance travelled by a frog for each jump during six consecutive jumps (Greenberg and Palen, 2021). Individuals were gently taken from their boxes and placed on the floor. In most cases, jumps occurred naturally, but on a few instances (i.e., for frogs from the 12 g L^−1^ group) jumping was elicited by gently probing the tip of the urostyle with a finger. Distance of each jump was measured with a ruler (±0.5 cm) and divided by the SVL of frogs to provide a more meaningful measure of locomotor performance (Van Damme and Van Dooren, 1999). The frogs were allowed to jump on an area of 9 m^2^ (3 × 3 m). The six consecutive jumps were measured by following the frog in its displacements. These assays were always performed at night, between 2 and 4 a.m., except for the measures made during recovery (after 12 h), which were performed between 2 and 4 p.m. Maximum jumping distances were also analysed and produced similar results than mean jumping distance (results not shown).

#### 2.4.2 Physiological Parameters

##### 2.4.2.1 Salt Fluxes and Osmolality

During all stages, the salinity of water was recorded daily (and each 12 h during the recovery period) to approximate the direction of diffusion of salt based on the change in water salinity, using a calibrated real-time conductivity meter (Testo 240, Testo AG, Germany). Plasma osmolality (mOsmol kg^−1^) was measured from 10 μl aliquots on a Vapro2 osmometer (Elitech group).

##### 2.4.2.2 Hemoglobin-Binding Protein

Individuals exposed to relatively high salt concentrations may present cells with ruptured membranes (Koleva et al., 2017), releasing free hemoglobin. Hemoglobin-binding proteins can be found in some amphibians species (in a newt species, *Taricha granulosa*, see Francis et al., 1985), but not in all (for example in *Xenopus tropicalis*, see Wicher and Fries, 2006 ) and are known to bind free hemoglobin in order to mitigate damages caused by reactive oxygen components during inflammation (Quaye, 2008; Andersen et al., 2017). We attempt to quantify these hemoglobin-binding proteins in 7.5 µL plasma samples using a commercially-available assay (TP801, Tri-Delta Diagnotics, United States). This colorimetric test measures the preservation of the peroxidase activity of bounded hemoglobin, which should be proportional to the concentration of the hemoglobin-binding proteins present in the plasma sample. We followed the instructions provided by the manufacturer with slight modifications following Matson et al. (2012). The standards, which were included in duplicate in each plate (*n* = 2), ranged from 2.5 to 0.039 mg ml^−1^. For 15 samples, values were below the standard curve and were therefore transformed to 0. The measured concentration of the hemoglobin-binding proteins varied from 0 to 2.45 mg ml^−1^. Using the standards, we calculated the intra- and inter-plate coefficients of variation to 2 and 4%, respectively. A negative (7.5 µl of diluent) control was also included in duplicate in each plate. We measured plasma absorbance (spectrophotometer Anthos 2010; Biochrom) at 600 nm, on 7.5 µl plasma, before adding the final reagent that induced the colorimetric reaction. Final absorbance values at 600 nm were corrected by the pre-scan absorbance values at 600 nm, which allowed us to control for sample differences in colour and cloudiness (Matson et al., 2012). We have to emphasize that this assay is still at is infancy in amphibians. The role of such proteins in these taxa is not well understood and further studies are needed in order to thoroughly describe hemoglobin-binding proteins in amphibians, and/or to develop a specific assay. Moreover, we used a kit aimed to measure binding activity to human hemoglobin. The affinity of frog’s proteins to human hemoglobin, used in this assay, is still unknown. As such, our assay could also have measured the peroxidase activity, instead of hemoglobin-binding proteins concentration.

##### 2.4.2.3 Leukocyte Counts

Neutrophils are potent effector cells of the innate immune response, which are relatively rapid and perform non-specific defences (Demas et al., 2011). In comparison, lymphocytes are implicated in the acquired immune response, and are generally slower and induce pathogen-specific responses that are an additional line of defence (Demas et al., 2011), even if they have innate-like properties (Flajnik, 2018). They play a key role in adaptive immunity, and can differentiate into memory cells and are involved in the production of antibodies (immunoglobin) (Du Pasquier et al., 1972; Sprent and Tough, 1994). A stress (i.e., a factor experienced by individuals beyond their tolerance range, that can decrease individuals fitness and/or increase mortality), such as salinity in our context, should modify the proportion of these leucocytes. Indeed, neutrophils and lymphocytes are affected by stress in opposite directions (Davis et al., 2008), as a stress may induce redistribution of lymphocytes from the blood to other body compartments (Dhabhar et al., 1995; Dhabhar, 2002; Davis et al., 2008), causing a significant reduction in their circulating numbers, while neutrophils are redirected from body compartments to the blood (Bishop et al., 1968; Davis et al., 2008). Thus, an increase in N:L ratio is considered to be a measure of an amphibian’s response to stress agents (Davis et al., 2008; Silva et al., 2020). In fact, high N:L ratios may indicate high levels of glucocorticoids (Davis et al., 2008), which alters leukocyte components in response to stress, even if not all organisms express a correlation between circulating glucocorticoid levels and N:L ratios, as these two variables play different roles in the stress response (Müller et al., 2011; Davis and Maney, 2018).

Monocytes are the precursors of tissue macrophages and act as cell phagocytes associated with innate defences against bacterial infections (van Furth and Cohn, 1968). They serve as phagocytic and antigen-presenting cells (Claver and Quaglia, 2009). Little is known about eosinophil function in amphibians, but there is some evidence that they respond to trematode infestations (Claver and Quaglia, 2009). Their increase is thought to be a common response to contaminants, as eosinophils respond to stimuli such as parasitic infections or pollutants (Kiesecker, 2002; Claver and Quaglia, 2009). The exact functions of basophils in amphibians have not yet been well-established (Claver and Quaglia, 2009), but they can participate in innate immune responses (by secreting proteins such as toxins) and serve as antigen-presenting cells (Demas et al., 2011). They also play an important role in early inflammation reactions (Claver and Quaglia, 2009).

To assess the effect of exposure to salt on leukocyte profile (see Burraco and Gomez-Mestre, 2016; Hall et al., 2020), a drop of blood was smeared on a slide. Slides were air-dried and fixed in absolute methanol for 5 min, and then coloured using May-Grünwald-Giemsa staining (3 min bath in May-Grunwald; 1 min bath in tampon solution, and 10 min bath in Giemsa 1/20). Neutrophils, lymphocytes (and the corresponding ratio of neutrophils to lymphocytes, hereafter N:L ratio), monocytes, eosinophils and basophils were counted by a single observer (LLS). Up to 100 leucocytes were counted, using a X100 ocular (Microscope Primo Star 8) microscope (total magnification ×1,000) and immersion oil.

Hemoglobin-binding proteins and leukocytes analyses were performed on 40 individuals (N = 120 samples: three blood samples per individuals, on 10 individuals per treatment). On these individuals, the four experimental groups had similar mass, size (SVL) and sex distribution (all *p*-values > 0.771; see Supplementary Appendix C).

### 2.5 Statistical Analysis

#### 2.5.1 Effects of Exposure to Salinity in the Field

To assess how salinity of the ponds influences ecological responses (sex ratio: proportion of females), morphology [SVL, mass, body condition index (BCI, calculated as the residuals from the regression of log body mass on log SVL)], physiology [osmolality, blood properties (hemoglobin-binding protein, N:L ratio, leukocyte profiles)], and behaviour [jumping performance, and activity (as activity is rated from 1 to 5, we evaluated it as a proportion)], we performed LMs (linear models) or quasibinomial GLMs (generalized linear models; for leukocytes profiles and activity) with salinity as continuous explanatory variable, or the quadratic form when models better fitted with a quadratic variation of salinity (using a backward selection procedure; likelihood ratio tests). As amphibians are sexually dimorphic (Wells, 2007), sex was also included as covariate in all models, as well as the interaction between salinity and sex. However, the effect of sex was never retained in the final models. Moreover, for sex ratio, we included Julian date as a covariate, but it was excluded during the model selection procedure. Finally, to understand if variations in jumping distance were influenced by individual morphology more than by pond salinity, we also computed LMs on jumping performance with SVL or hindlimb length as covariate. Mean jumping distance was not linked to body size (Estimate = 0.059, SE = 0.039, t-value = 1.513, *p*-value = 0.132) or hindlimb length (Estimate = 0.048, SE = 0.048, t-value = 1.018, *p*-value = 0.31).

#### 2.5.2 Effects of Experimental Exposure to Salinity

##### 2.5.2.1 Variations During Acclimation

Variations in all the studied parameters (body mass, osmolality, N:L ratio, leucocyte proportions, hemoglobin-binding protein concentration, jumping performance and activity) during acclimation were assessed using LMMs (linear mixed models) or quasibinomial GLMMs (generalized linear mixed models), and Tukey post-hoc tests. Time was set as an explanatory variable, and individual as a random effect. Differences between experimental groups after acclimation were assessed using LMs and quasibinomial GLMs with treatment as a covariate, with Tukey post-hoc tests.

##### 2.5.2.2 Variations During Exposure and Recovery

The effect of salinity was assessed using four salinity treatments (0, 6, 9 and 12 g L^−1^).

The effect of each treatment was assessed on mass (using absolute body mass changes, values at 48 h exposure compared to the beginning of exposure for variations during exposure, values at 24 h recovery compared to the end of exposure for variations during recovery). These effects were also assessed on changes in the concentration of the treatment (using variation in water concentration, values at 48 h exposure compared to the beginning of the exposure), and on changes in concentration of the recovery treatment (using variation in water concentration, values at 24 h recovery compared to the beginning of the recovery). Finally, the effect of each treatment was assessed on osmolality, hemoglobin-binding protein and the N:L ratio of individuals (at 48 h exposure compared to the beginning of the exposure), and on jumping performance (corrected by individuals size, at 48 h exposure compared to the beginning of exposure for variations during exposure, and at 24 h recovery compared to the end of exposure for variations during recovery). All these effects were assessed using LMMs.

The effect of each treatment on the leukocyte profiles at 48 h treatment, and on activity (as activity is rated from 1 to 5, we evaluated it as a proportion; at 48 h exposure compared to the beginning of exposure for variations during exposure, and at 24 h recovery compared to the end of exposure for variations during recovery), was assessed using quasibinomial GLMMs.

In all of these models, we included treatment, time, pond salinity, sex and SVL as covariates, as well as the interactions between treatment and time, treatment and pond salinity, treatment and SVL and treatment and sex as covariates. As we performed multiple measures on a single individual, individual was set as a random effect. The best model was selected using a backward selection procedure (0.1 cut-off *p*-value) using likelihood-ratio tests, and only the retained parameters were represented. The effects of pond salinity and sex were never retained in the selection procedure. Each model performed were checked for accuracy by analyzing residuals distribution and over/under dispersion.

In order to facilitate comprehension, the intermediate measures (at 24 h exposure and 12 h recovery) that were assessed for some variables (i.e., body mass and changes in treatment salinity at 24 h exposure, and changes in salinity and jumping distance at 12 h recovery), are not presented in results, but are represented in Supplementary Appendix D for treatment and Supplementary Appendix E for recovery. Statistics were computed and gave the same results as when we compared only the end of the treatment and the end of the recovery. Detailed statistics are also presented in Supplementary Appendix F for treatment, and Supplementary Appendix G for recovery.

#### 2.5.3 Mortality

Finally, to understand determinants of mortality during our treatments, we computed GLMs with quasibinomial distribution and mass, loss in mass during the treatment, size (SVL), BCI, sex, salinity of the pond and osmolality as covariates. We did not include salinity of treatments, as only eight individuals from the 12 g L^−1^ treatment died. Because of this low sample size, we did not compute a complete model with all these variables together, and all analyses for these different covariates were conducted separately.

All data analysis were performed using R 3.6.3 (R Core Team, 2020) and Rstudio v1.1.419.

## 3 Results

### 3.1 Effects of Exposure to Salinity in the Field

#### 3.1.1 Sex-Ratio and Morphology

**FIGURE 1:**
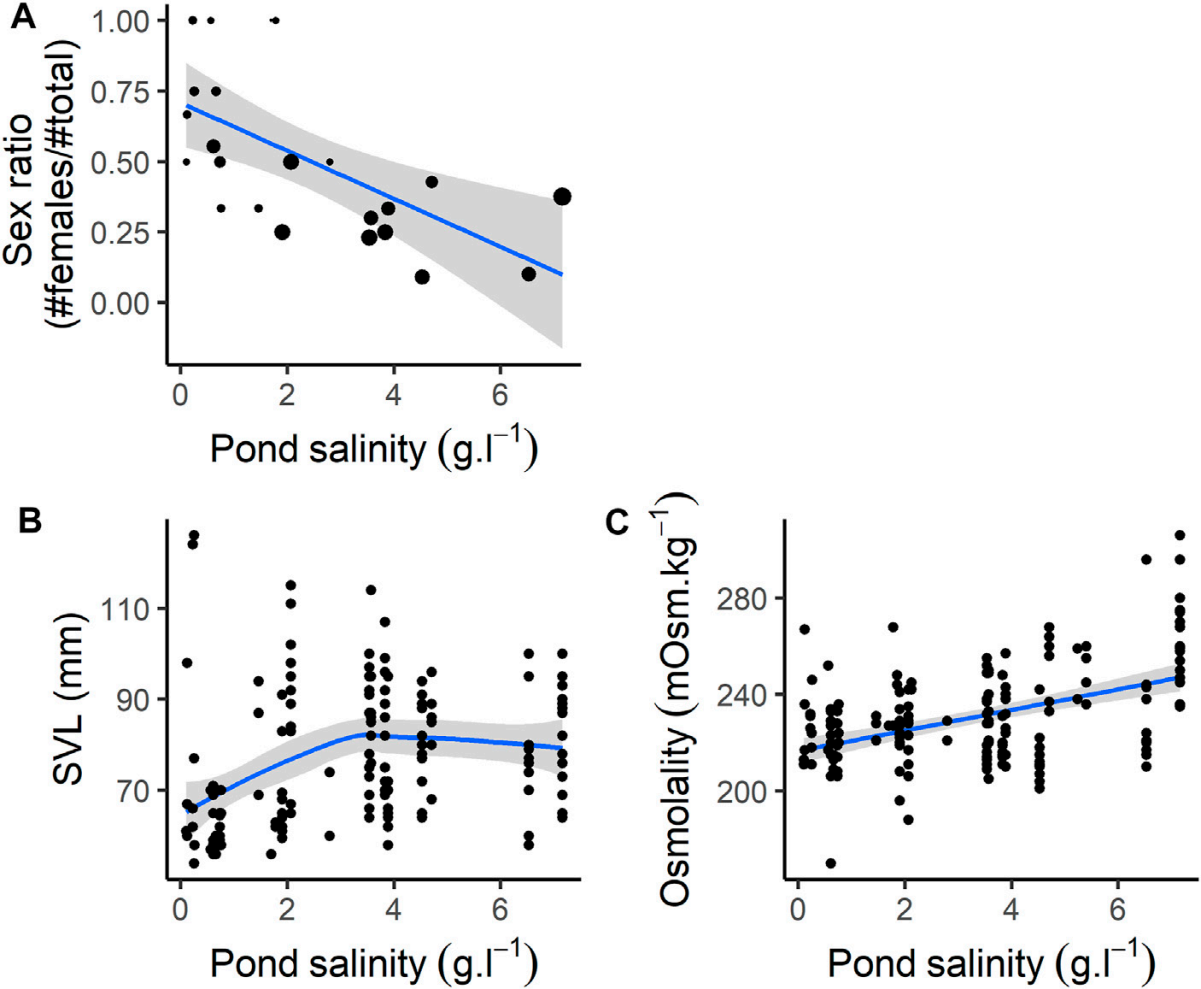
Individual characteristics according to pond salinity. **(A)** Sex ratio (note that size of the dots are proportional to the sample size, ranging from 1 to 16, see Supplementary Appendix B), **(B)** SVL (size, mm), **(C)** Osmolality.

Sex ratio (proportion of females compared to total number of individuals) decreased with pond salinity (Figure 1A; Table 1). Individuals were larger in ponds with higher salinity (Figure 1B; Table 1), and heavier, as body mass increased with increasing salinity (Table 1). BCI was lower in ponds with higher salinity (Table 1).

**TABLE 1:** Effect of pond salinity on osmolality, SVL (size, mm), mass (g), body condition (BCI), Sex ratio, hemoglobin-binding protein, Neutrophils/Lymphocytes ratio (N:L ratio), leucocytes counts, jumping distance (as the jumping distance corrected by individuals SVL), and activity. Computed on N = 156 individuals (see also Supplementary Appendix B for number of individuals captured on each site), except for hemoglobin-binding proteins and leucocytes counts, where it was computed on N = 40 individuals.

Comparison	Covariable	*R* ^2^	F	Estimate	SE	t/z	*p*-value
Sex ratio (#Females/#Total)	Salinity	-	-	−0.276	0.085	−3.236	0.001
SVL	Salinity	0.11	10.61	7.087	1.859	3.811	<0.001
	Salinity^2^	0.11	10.61	−0.714	0.246	−2.897	0.004
Mass	Salinity	0.008	2.194	1.653	1.116	1.481	0.141
BCI	Salinity	0.218	22.62	0.016	0.004	3.559	<0.001
	Salinity^2^	0.218	22.62	−0.003	0.001	−5.062	<0.001
Osmolality	Salinity	0.194	40.81	4.269	0.668	6.388	<0.001
Neutrophil proportion	Salinity	-	-	−0.019	0.036	−0.544	0.59
Lymphocyte proportion	Salinity	-	-	0.002	0.015	0.145	0.886
N:L ratio	Salinity	−0.025	0.053	−0.003	0.012	−0.23	0.819
Monocyte proportion	Salinity	-	-	−0.088	0.04	−2.178	0.036
Eosinophil proportion	Salinity	-	-	0.136	0.063	2.144	0.039
Basophil proportion	Salinity	-	-	0.066	0.126	0.527	0.601
Hemoglobin-binding protein concentration	Salinity	0.007	1.281	−0.011	0.01	−1.132	0.265
Jumping performance	Salinity	0.157	15.48	−0.871	0.16	−5.446	<0.001
	Salinity^2^	0.157	15.48	0.103	0.021	4.881	<0.001
Activity	Salinity	-	-	<0.001	<0.001	−0.018	0.985

#### 3.1.2 Physiological Parameters

Osmolality increased with pond salinity (Figure 1C; Table 1). Neither the proportion of neutrophils, lymphocytes and basophils, nor the N:L ratio were correlated with pond salinity (Table 1). The proportion of monocytes decreased, while the proportion of eosinophils increased, with increasing pond salinity (Table 1). Details on cell counts from blood smears are given in Supplementary Appendix H. Hemoglobin-binding protein concentration was not correlated with pond salinity (Table 1).

#### 3.1.3 Jumping Performance and Activity

Mean jumping distance was correlated with pond salinity with a curvilinear relationship (Table 1). Jumping distance decreased with pond salinity <∼4 g L^−1^, and increased with pond salinity >∼4 g L^−1^. Activity scores were not correlated with pond salinity (Table 1).

### 3.2 Effects of Experimental Exposure to Salinity

The effects of acclimation in freshwater are summarized in Supplementary Appendix I. At the onset of the experimental exposure to salinity, all groups were similar for all the parameters measured (Supplementary Appendix I), except for osmolality (Supplementary Appendix I), where values for individuals exposed to 12 g L^−1^ were slightly higher compared to those exposed to 6 g L^−1^ (Estimate = 9.684, SE = 3.356, z-value = 2.885, *p*-value = 0.023).

#### 3.2.1 Body Mass

**FIGURE 2:**
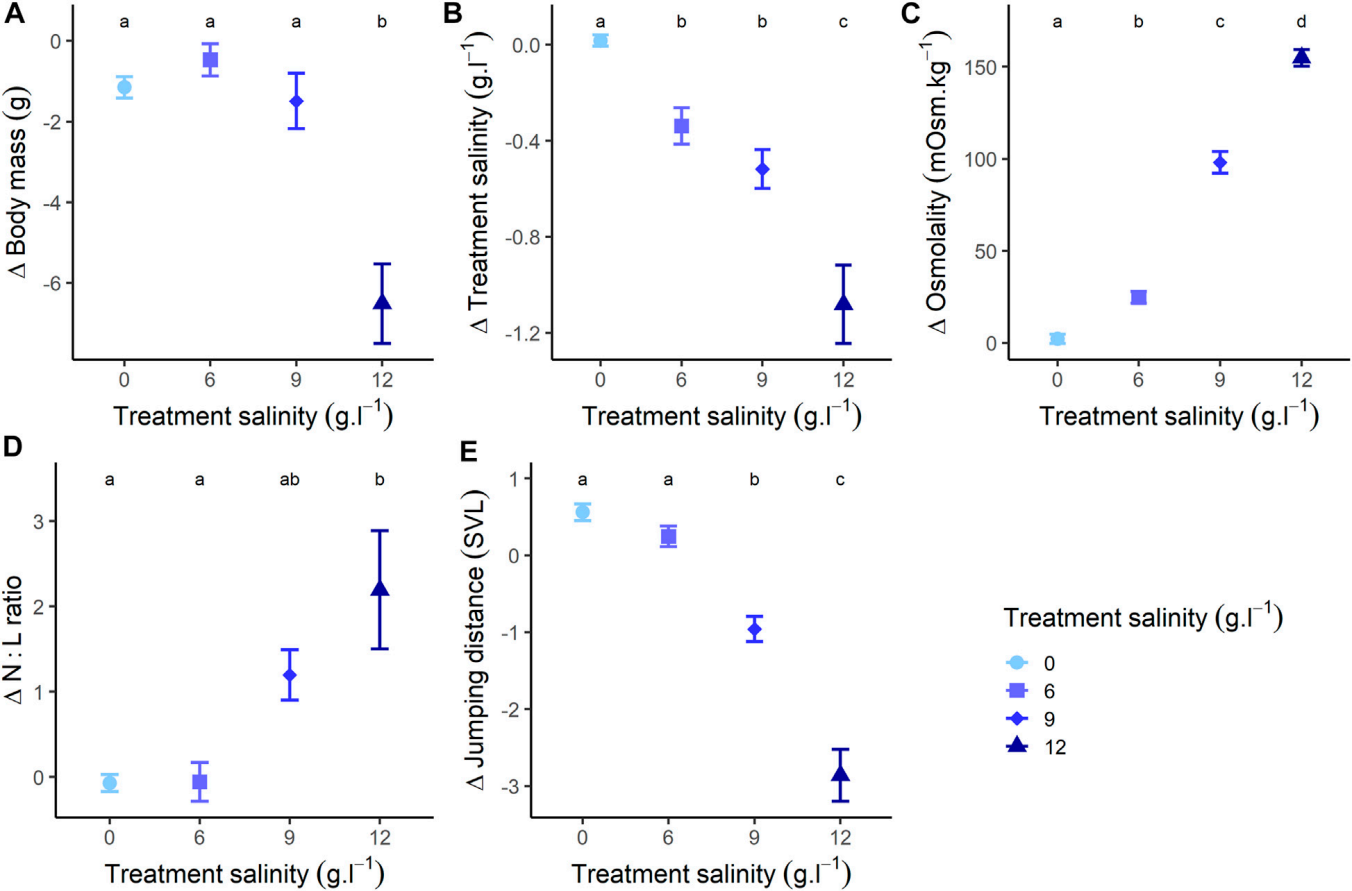
Changes of **(A)** body mass, **(B)** salinity (changes in treatment salt concentration during exposure), **(C)** osmolality, **(D)** N:L ratio (Neutrophils/Lymphocytes ratio), and **(E)** jumping performance (relative to individuals body size: mean distance [mm]/individuals body size [mm]), at the end of the experiment (48 h treatment) compared to the beginning of the experiment. Different letters represent a significative difference at α = 0.05 for variations during exposure.

The changes in body mass during exposure were different between treatments (Figure 2A; Supplementary Appendix J).

Individuals from the freshwater group showed a slight mass loss during exposure (Estimate = −1.151, SE = 0.430, z-value = −2.677, *p*-value = 0.021) (Figure 2A; Supplementary Appendix F). Individuals exposed to 6 g L^−1^ showed a relatively stable pattern with no mass changes (Estimate = −0.470, SE = 0.435, z-value = −1.080, *p*-value = 0.527) (Figure 2A; Supplementary Appendix F). Individuals exposed to 9 g L^−1^ lost mass during exposure (Estimate = −1.490, SE = 0.538, z-value = −2.768, *p*-value = 0.016) (Figure 2A; Supplementary Appendix F), as well as individuals exposed to 12 g L^−1^ (Estimate = −6.270, SE = 0.537, z-value = −11.666, *p*-value < 0.001) (Figure 2A; Supplementary Appendix F). At the end of the exposure, body mass was similar between individuals exposed to 0, 6 and 9 g L^−1^ (all *p*-values > 0.277), but significantly lower for individuals at 12 g L^−1^ (all *p*-values < 0.004) (Figure 2A). The body size of individuals did not influence the mass changes across treatments (Supplementary Appendix J).

#### 3.2.2 Salt Influx and Osmolality

The changes in salt concentration of water during exposure were different between treatments (Figure 2B; Supplementary Appendix J). In the freshwater group, treatment concentration remained constant during exposure (*p*-value = 0.973) (Figure 2B; Supplementary Appendix F). In the 6 g L^−1^ treatment (Estimate = −0.338, SE = 0.079, z-value = −4.274, *p*-value < 0.001), the 9 g L^−1^ treatment (Estimate = −0.518, SE = 0.097, z-value = −5.317, *p*-value < 0.001) and the 12 g L^−1^ treatment (Estimate = −1.069, SE = 0.098, z-value = 10.936, *p*-value < 0.001), concentration decreased during exposure (Figure 2B; Supplementary Appendix F). At the end of the exposure, changes in concentration were similar for the 6 g L^−1^ and 9 g L^−1^ treatments (*p*-value = 0.448), but significantly lower for the 0 g L^−1^ treatment and higher for the 12 g L^−1^ treatment (all *p*-values < 0.005) (Figure 2B). The body size of individuals did not influence the changes of treatment concentration across treatments (Supplementary Appendix J).

Accordingly with the observed diverging salt influxes, the changes in plasma osmolality values during exposure were different between treatments (Figure 2C; Supplementary Appendix J). Osmolality of individuals in the freshwater group remained stable during the whole experiment (Estimate = 2.722, SE = 3.457, z-value = 0.739, *p*-value = 0.711) (Figure 2C; Supplementary Appendix F). Conversely, osmolality increased in individuals exposed to 6 g L^−1^ (Estimate = 24.781, SE = 3.158, z-value = 7.114, *p*-value < 0.001), 9 g L^−1^ (Estimate = 98.067, SE = 5.512, z-value = 23.350, *p*-value < 0.001) and 12 g L^−1^ (Estimate = 153.839, SE = 3.659, z-value = 35.372, *p*-value < 0.001) (Figure 2C; Supplementary Appendix F). As a consequence, osmolality was different across all groups at the end of the exposure (all *p*-value < 0.001). Additionally, variations of body mass were negatively correlated with osmolality (Estimate = −0.027, SE = 0.005, t-value = −5.480, *p*-value < 0.001), and change in treatment concentration (i.e., the salt concentration of their housing water) were negatively correlated with osmolality (Estimate = −0.003, SE < 0.001, t-value = −5.783, *p*-value < 0.001). The body size of individuals did not influence the osmolality changes across treatments (Supplementary Appendix J).

#### 3.2.3 Leukocytes Counts

The changes in lymphocyte proportion during exposure were different between salinity treatments (Supplementary Appendix J). More specifically, lymphocyte proportion remained stable for individuals in the freshwater group (*p*-value = 0.842), but increased for individuals exposed to 6 g L^−1^ (*p*-value = 0.032), and decreased for individuals exposed to 9 g L^−1^ (Estimate = −0.488, SE = 0.079, z-value = −6.166, *p*-value < 0.001) and 12 g L^−1^ (Estimate = −0.775, SE = 0.087, z-value = −8.895, *p*-value < 0.001). At the end of the exposure, lymphocyte proportions were similar between individuals exposed to 0 and 6 g L^−1^ (all *p*-values > 0.102), but significantly lower for individuals exposed to 9 g L^−1^ (all *p*-values < 0.026) and 12 g L^−1^ (all *p*-values < 0.016).

The changes in neutrophil proportion during exposure were different between salinity treatments (Supplementary Appendix J). Neutrophil proportions remained constant in the 0 g L^−1^ (*p*-value = 0.662) and 6 g L^−1^ (*p*-value = 0.218) treatments but increased in the 9 g L^−1^ (Estimate = 0.838, SE = 0.095, z-value = 8.825, *p*-value < 0.001) and 12 g L^−1^ (Estimate = 0.969, SE = 0.094, z-value = 10.312, *p*-value < 0.001) treatments. At the end of the exposure, neutrophil proportions were similar for individuals exposed to 12 g L^−1^ and 9 g L^−1^ (*p*-value = 0.813), and for individuals exposed to 0 g L^−1^ and 6 g L^−1^ (*p*-value = 0.710). All other comparisons were significant (all *p*-values < 0.001).

As a consequence, during exposure, N:L ratios varied differently across treatments (Figure 2D; Supplementary Appendix J). This ratio remained stable for individuals in the freshwater group (*p*-value = 0.859) or exposed to 6 g L^−1^ (*p*-value = 0.886), but increased for individuals exposed to 9 g L^−1^ (Estimate = 1.197, SE = 0.397, z-value = 3.012, *p*-value = 0.005) and 12 g L^−1^ (Estimate = 2.195, SE = 0.397, z-value = 5.522, *p*-value < 0.001) (Figure 2D; Supplementary Appendix F). At the end of the exposure, N:L ratios were similar for individuals exposed to 9 g L^−1^ and 12 g L^−1^ (*p*-value = 0.054) and for individuals exposed to 0 g L^−1^ and 6 g L^−1^ (*p*-value = 0.997), but the other comparisons were significant (all *p*-values < 0.037).

The changes in eosinophil proportion during exposure were different between salinity treatments (Supplementary Appendix J). Eosinophil proportions remained constant for individuals exposed to 0 g L^−1^ (*p*-value = 0.224) and 6 g L^−1^ (*p*-value = 0.567), but decreased for individuals exposed to 9 g L^−1^ (Estimate = −0.858, SE = 0.247, z-value = −3.470, *p*-value < 0.001) and 12 g L^−1^ (Estimate = −1.101, SE = 0.273, z-value = −4.035, *p*-value < 0.001). Overall, at the end of exposure, eosinophil proportions did not differ between treatments (for all *p*-value > 0.101), except for individuals exposed to 0 g L^−1^ which had a higher eosinophil proportions compared to those exposed to 12 g L^−1^ (*p*-value = 0.016).

Conversely, monocyte proportions and basophil proportions did not vary through time and treatment during the experiment (both *p*-value > 0.314) (Supplementary Appendix J). Additionally, the concentration of hemoglobin-binding proteins did not vary through time and treatment during the experiment (*p*-value = 0.520) (Supplementary Appendix J). For all blood cell counts and hemoglobin-binding protein, the body size of individuals did not influence their response to exposure to salinity across treatments (Supplementary Appendix J).

#### 3.2.4 Behaviour

The changes in jumping distance during exposure varied among treatments (Figure 2E; Supplementary Appendix J). Individuals from the freshwater group showed an increase in jumping distance during exposure (Estimate = 0.561, SE = 0.158, z-value = 3.547, *p*-value < 0.001), and individuals exposed to 6 g L^−1^ showed no variation in jumping distance (Estimate = 0.246, SE = 0.160, z-value = 1.539, *p*-value = 0.126) (Figure 2E; Supplementary Appendix F). Conversely, jumping distance decreased for individuals exposed to 9 g L^−1^ (Estimate = −0.958, SE = 0.198, z-value = −4.838, *p*-value < 0.001), and 12 g L^−1^ (Estimate = −2.901, SE = 0.203, z-value = −14.313, *p*-value < 0.001) (Figure 2E; Supplementary Appendix F). At the end of the exposure, jumping distance was similar between individuals exposed to 0 and 6 g L^−1^ (*p*-value = 0.957), but significantly lower for individuals in other treatments (for all, *p*-value < 0.031) (Figure 2E). There was an effect of size as, for all treatments, jumping performance decreased more for larger individuals (Estimate = −0.050, SE = 0.005, t-value = −9.317, *p*-value < 0.001) (Supplementary Appendix J).

The changes in activity during exposure were also different among treatments (Supplementary Appendix J). Individuals exposed to 0 g L^−1^ (*p*-value = 0.956), 6 g L^−1^ (*p*-value = 0.998) and 9 g L^−1^ (*p*-value = 0.836) showed stable activity scores during exposure. Conversely, activity scores of individuals exposed to 12 g L^−1^ decreased (Estimate = −0.860, SE = 0.181, z-value = −4.750, *p*-value < 0.001). At the end of the exposure, activity scores were similar between individuals exposed to 0, 6 and 9 g L^−1^ (all *p*-values > 0.999), but significantly lower for individuals at 12 g L^−1^ (all *p*-values < 0.001). The body size of individuals did not influence the activity changes across treatments (Supplementary Appendix J).

### 3.3 Effects of Recovery in Freshwater

#### 3.3.1 Body Mass

**FIGURE 3:**
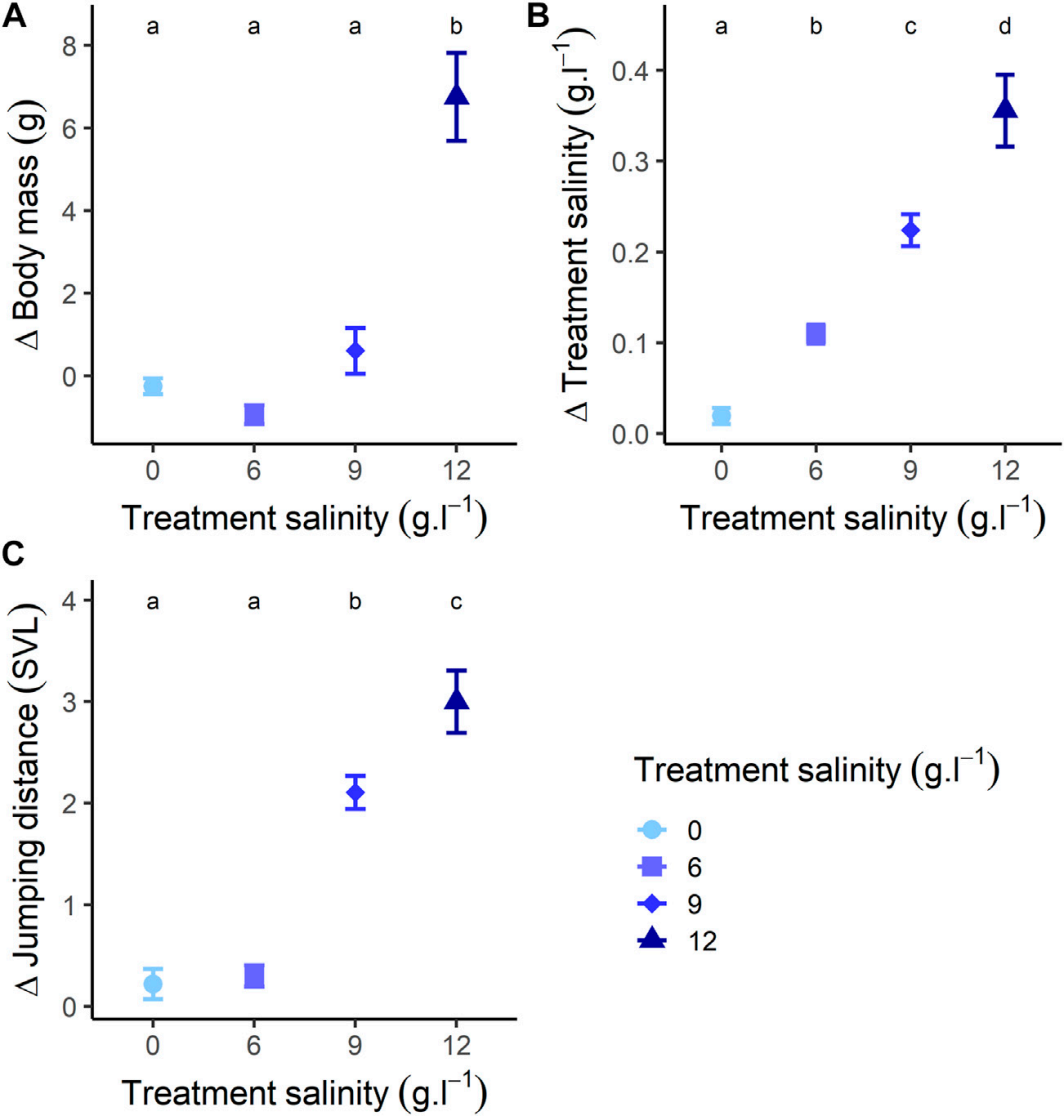
Changes of **(A)** body mass, **(B)** salinity (changes in salt concentration during recovery), and **(C)** jumping performance (relative to individuals body size: mean distance [mm]/individuals body size [mm]), at the end of the recovery (24 h recovery) compared to the beginning of the recovery. Different letters represent a significative difference at α = 0.05 for variations during recovery.

The changes in body mass during recovery were different across treatments (Figure 3A; Supplementary Appendix J). Individuals previously exposed to 0 and 9 g L^−1^ treatments showed a stable pattern during recovery (for both *p*-value > 0.240) (Figure 3A; Supplementary Appendix G). On the contrary, body mass of individuals previously exposed to 6 g L^−1^ significantly decreased (Estimate = −0.947, SE = 0.432, z-value = −2.191, *p*-value = 0.030) (Figure 3A; Supplementary Appendix G). Conversely, the body mass of individuals previously exposed to 12 g L^−1^ significantly increased (Estimate = 6.661, SE = 0.564, z-value = 11.812, *p*-value < 0.001) (Figure 3A; Supplementary Appendix J) and attained body mass similar to that of the beginning of the exposure (Estimate = 0.202, SE = 0.589, z-value = 0.344, *p*-value = 0.732) (Figure 3A). At the end of the recovery, body mass was similar among treatments (for all *p*-value > 0.059) (Figure 3A). We found an effect of body size on the pattern of body mass gain only in individuals exposed to 12 g L^−1^ (Supplementary Appendix J). In this group, larger individuals gained more mass (Estimate = 0.143, SE = 0.067, t-value = 2.150, *p*-value = 0.042) (Supplementary Appendix J).

#### 3.3.2 Salt Efflux

The changes in salt concentration of water during recovery were different among treatments (Figure 3B; Supplementary Appendix J). Salt concentration remained stable for individuals previously exposed to 0 g L^−1^ (*p*-value = 0.390) (Figure 3B; Supplementary Appendix J). Conversely, salt concentration increased for individuals previously exposed to 6, 9 and 12 g L^−1^ (for all *p*-values < 0.001) (Figure 3B; Supplementary Appendix G). After 24 h recovery, changes in salt concentration were different among all treatments (all *p*-values < 0.001) (Figure 3B). We found an effect of body size on the pattern of variation in salt concentration in individuals previously exposed to 6 g L^−1^ (Estimate = 0.002, SE < 0.001, t-value = 3.486, *p*-value = 0.001), 9 g L^−1^ (Estimate = 0.004, SE < 0.001, t-value = 5.181, *p*-value < 0.001) and 12 g L^−1^ (Estimate = 0.010, SE = 0.001, t-value = 6.200, *p*-value < 0.001) (Supplementary Appendix J). Increase in salt concentration was higher for larger individuals.

#### 3.3.3 Behaviour

The changes in jumping distance during recovery were different across treatments (Figure 3C; Supplementary Appendix J). Jumping distance of individuals previously exposed to 0 g L^−1^ remained stable (Estimate = −0.251, SE = 0.138, z-value = −1.822, *p*-value = 0.164) (Figure 3C; Supplementary Appendix G). Jumping distance increased for individuals previously exposed to 6 g L^−1^ (Estimate = 0.331, SE = 0.135, z-value = 2.453, *p*-value = 0.039) (Figure 3C; Supplementary Appendix G). Jumping distance of individuals previously exposed to and 9 g L^−1^ increased (Estimate = 2.108, SE = 0.162, z-value = 13.015, *p*-value < 0.001), and reached values greater than those recorded at the beginning of the exposure (Estimate = 1.150, SE = 0.178, z-value = 6.446, *p*-value < 0.001) (Figure 3C; Supplementary Appendix G). Similarly, jumping distance of individuals previously exposed to 12 g L^−1^ increased (Estimate = −2.993, SE = 0.183, z-value = −16.339, *p*-value < 0.001), and became similar to jumping performance at the beginning of exposure (*p*-value = 0.999) (Figure 3C; Supplementary Appendix G). After 24 h recovery, jumping distance was similar among individuals previously exposed to 0, 6 and 12 g L^−1^ (all *p*-values > 0.545), but significantly higher for individuals at 9 g L^−1^ (all *p*-values < 0.012) (Figure 3C). There was an effect of size where, for all treatments, the increase in jumping performance was lower for larger individuals (Estimate = −0.061, SE = 0.009, z-value = −6.905, *p*-value < 0.001) (Supplementary Appendix J).

The changes in activity during recovery were different among treatments (Supplementary Appendix J). Individuals previously exposed to 0 g L^−1^ (*p*-value = 0.992), 6 g L^−1^ (*p*-value = 0.991) and 9 g L^−1^ (*p*-values = 0.977) did not vary in activity scores during recovery. Conversely, activity scores of individuals previously exposed to 12 g L^−1^ increased (Estimate = 0.827, SE = 0.196, z-value = 4.226, *p*-value < 0.001). Overall, activity scores between the beginning of the treatment and the end of the recovery were similar (*p*-value = 0.981). At 24 h recovery, activity was similar among all groups (all *p*-values > 0.993). The body size of individuals did not influence the activity changes across treatments (Supplementary Appendix J).

The overall effects of salinity (both in the field and at the lab) on morphological, physiological and behavioural parameters are summarized in Table 2.

**TABLE 2:** Summary of the effects of increasing salinity on morphological, physiological and behavioural parameters (both in the field and experimentally). NS, Non-significant, - indicates variables that have not been tested, computed on N = 156 individuals (0 g L^−1^: N = 47; 6 g L^−1^: N = 46; 9 g L^−1^: N = 30; 12 g L^−1^: N = 33), except for hemoglobin-binding proteins and leucocytes counts, where it was computed on N = 40 individuals (10 per treatments).

Explanatory variables	Effects of increasing salinity		
	Field (0.10–7.16 g L^−1^)	Experimental	
		Exposure (0–12 g L^−1^)	Recovery
Sex ratio (#Females/#Total)	Decrease	-	-
SVL	Increase	-	-
Mass	NS	Decrease	Increase
BCI	Decrease	-	-
Treatment concentration	-	Decrease	Increase
Osmolality	Increase	Increase	-
Lymphocyte proportion	NS	Decrease	-
Neutrophil proportion	NS	Increase	-
N:L ratio	NS	Increase	-
Eosinophil proportion	Increase	Decrease	-
Monocyte proportion	Decrease	NS	-
Basophil proportion	NS	NS	-
Hemoglobin-binding protein concentration	NS	NS	-
Jumping performance	Increase for intermediate salinities (4 g L^−1^)	Decrease	Increase
Activity	NS	Decrease	Increase

### 3.4 Mortality

During our experiments, eight individuals died after being exposed to the 12 g L^−1^ treatment: five during treatment, three during recovery. Mortality was linked to morphology and occurred in smaller and lighter individuals (Table 3).

**TABLE 3:** Effects of mass, loss of mass, SVL (size), body condition, sex, pond salinity and osmolality on individual mortality in the 12 g L^−1^ salinity treatment (computed only on individuals exposed to the 12 g L^−1^ salinity treatment, N = 33 individuals).

Covariate	Estimate	SE	*t*-value	*p*-value
Mass	−0.145	0.06	−2.4	0.023
Loss in mass	0.124	0.092	1.353	0.186
SVL	−0.141	0.064	−2.199	0.028
BCI	−10.432	11.94	−0.874	0.382
Sex	−0.065	0.841	−0.077	0.939
Pond salinity	0.371	0.192	1.934	0.053
Osmolality	−0.045	0.047	−0.962	0.336

## 4 Discussion

Our study showed that the physiology and behaviour of a coastal frog are influenced by environmental levels of salinity, both in the field and experimentally. Our study species can be found in ponds in which the salinity (ranging from 0 to 16 g L^−1^) is similar to those experienced by other species found in coastal marshes (Hopkins and Brodie, 2015). The salinity experienced in the field (0–7 g L^−1^) was correlated with physiological parameters (osmolality, monocytes and eosinophils counts), negatively correlated with body condition and locomotor performance and seems to influence size- and sex-specific habitat selection (see also Lorrain-Soligon et al., 2022b). Experimentally, we demonstrated that short-term exposure to environmental salinity significantly affects physiological parameters [salt influxes (osmolality), water effluxes (body mass), immunity (leukocyte counts)] and locomotor performance (jumping distances and activity). Recovery in freshwater indicates that most of these effects were transient (water and salt effluxes, locomotor performance). Taken together, these results suggest that salinity can have important, but transient, effects on behaviour and physiology even in a species thought to be salt-tolerant (Natchev et al., 2011; Mollov, 2020).

### 4.1 Insights From Field Investigations

In the field, individuals from brackish ponds were larger but lighter, had higher osmolality and had lower jumping performance than their counterparts from freshwater sites. Such results suggest that brackish ponds are colonized by larger individuals, presumably because smaller individuals have a larger skin surface area to volume ratio, which could increase transcutaneous rates of water loss and salt gain (Gordon et al., 1961) thereby limiting their ability to remain in brackish sites. Yet, individuals from brackish ponds were lighter and had lower locomotor performance and several complementary and non-mutually exclusive hypotheses can explain such results. First, it is possible that the effects of elevated salinity on locomotor performance (Squires et al., 2008; Denoël et al., 2010; Wood and Welch, 2015; Hall et al., 2017) influence foraging efficiency, thereby decreasing energy gain and thus decreasing body condition. Second, lower body condition in individuals from brackish sites may be a mere consequence of dehydration (water effluxes) linked to contact with brackish water (Shoemaker and Nagy, 1977; Gonzalez, 2012; Brischoux et al., 2017; Lorrain-Soligon et al., 2022a). Third, osmoregulation can be energetically costly (Kidder et al., 2006; Rivera-Ingraham and Lignot, 2017) and individuals living in brackish water may experience higher energy expenditure, thereby reducing their body condition (De Boeck et al., 2000). Teasing apart the relative contribution of these complementary processes deserves further investigation.

Interestingly, more males were located in brackish sites than females. These results are relatively consistent with the processes highlighted above. Indeed, adult females mobilize large amount of energy to reproduce (Wells, 2007; Hayward and Gillooly, 2011) and energetic constraints of life in brackish water (either directly linked to osmoregulation, or indirectly linked to foraging efficiency, see above) may negatively interact with the energetic requirements of reproduction. Complementarily, such a biased sex-ratio may also be linked to habitat selection processes for which amphibian females have been shown to select less brackish sites to mate and lay their eggs in order to decrease the negative consequences of elevated salinity on embryonic and larval development (Haramura, 2008; Albecker and McCoy, 2017; Lorrain-Soligon et al., 2022b). It is noteworthy that we focused our investigation on pond salinity and we did not assess other parameters. Future studies should usefully investigate the effects of such environmental characteristics in relation with salinity.

Neither the proportions of neutrophils, lymphocytes nor basophils were influenced by pond salinity. However, the proportion of monocytes decreased, while the proportion of eosinophils increased, with increasing pond salinity. Monocytes act as cell phagocytes associated with innate defences against bacterial infections (Davis et al., 2008), and eosinophils respond to stimuli such as parasitic infections or pollutants (Kiesecker, 2002; Claver and Quaglia, 2009). As such, it seems that salinity could influence leukocyte profiles in the sampled ponds. One hypothesis could be that salinity differentially affected the different leukocytes (Burraco and Gomez-Mestre, 2016). Indeed, increased salinity is thought to exert immunosuppressive effects (Gutiérrez et al., 2013; Gutiérrez, 2014). Alternatively, salinity could influence the type and/or the frequency of the encountered pathogens. For instance, in one amphibian genus (two species; *Litoria aurea* and *Litoria fallax*), it has been shown that *Batrachochytrium* infection and transmission is reduced with increasing salinity (White, 2006; Stockwell et al., 2015; Clulow et al., 2018). In birds, it has been shown that a relatively lower diversity of blood parasites is observed in saline habitats because of a reduced abundance in invertebrate vectors (Yohannes et al., 2009; Gutiérrez, 2014). Thus, the observed differences in leukocyte profiles could reflect changes in the prevalence and virulence of pathogens and parasites with salinity. To tease apart these two hypotheses, parasites and pathogens need to be sampled in individuals to understand if the change in leukocyte proportion is a consequence of a shift in parasites and pathogens presence and/or virulence with increasing salinity. Hemoglobin-binding protein did not covary with salinity. This suggests that damage to blood cells (and thus the presence of free hemoglobin) due to salinity, as highlighted in some species (Koleva et al., 2017), does not occur in coastal frogs. Nevertheless, it is important to note that the maximum salinity in which we captured individuals remains moderate (7 g L^−1^) and it is possible that exposure to higher salinity may have induced greater effects (see below).

### 4.2 Insights From Experimental Investigations

#### 4.2.1 Water and Salt Influx and Osmolality

The main physiological consequence linked to exposure to salinity that we observed was an increase in the osmotic concentration of body fluids (Brischoux and Kornilev, 2014; Lukens and Wilcoxen, 2020; Park and Do, 2020; Nagy et al., 2021; Tornabene et al., 2022) and a concomitant increase in water loss (Gonzalez, 2012). The increase in osmolality for individuals in saline treatments is linearly related to water salinity, and reveals salt influxes (Licht et al., 1975; Brischoux and Kornilev, 2014; Nagy et al., 2021). This linearity suggests that salt intake occurs through passive mechanisms, a process that resemble osmoconformity (i.e., passive diffusion of ions according to the osmolality of the environment, see also Weick and Brattstrom, 2021). Such a result is strengthened by the fact that water salinity—and not body size, sex or salinity of the pond of origin—affected osmolality. Importantly, we also measured a significant decrease in the salinity of the water during the experimental treatment. Such a result indicates that individuals have gained salt from the water (as corroborated by an increase in osmolality) and/or have diluted the treatment water due to water loss (as corroborated by a decrease in body mass in some treatments, Brischoux et al., 2017). Interestingly, body mass loss was observed for the highest salinity tested (12 g L^−1^), suggesting that contrarily to salt gain, water loss occurred only in the high salinity treatment. Such a result further indicates that mechanisms that aim at limiting water loss are at play (e.g., electrolyte production, Gordon 1965; Park and Do 2020), and that these mechanisms are efficient at limiting water loss in amphibians, at least up to 9 g L^−1^. Finally, during recovery in freshwater, we found the opposite responses. Although we did not collect blood at the end of this stage, salt concentration in the water during the recovery stage of our experiment increased linearly with the salinity of the preceding treatment, suggesting that individuals flushed excess salt passively according to the osmotic gradient. In addition, water absorption (body mass gain) occurred in individuals previously exposed to the 12 g L^−1^ treatment (Hillyard et al., 1998). Collectively, these results suggest that ion and water balance equilibration may occur rapidly when freshwater is available (Shoemaker and Nagy, 1977).

#### 4.2.2 Immunological Changes

It has been observed in different amphibian species that exposure to high salinity increases the susceptibility of tadpoles to infection (Milotic et al., 2017; Hall et al., 2020). In addition, salt-exposed amphibians exhibit stress-induced leukocyte responses (Davis et al., 2008). We show that individuals exposed to a high salinity treatment express a greater N:L ratio, driven by a decrease in the proportion of lymphocytes and an increase in the proportion of neutrophils. This process has already been highlighted as a response to high salinity, in larval amphibians (Burraco and Gomez-Mestre, 2016) and terrapin (Ashley et al., 2021). Although we did not assayed glucocorticoids in the current study, it is noteworthy that such response may be a corticosterone-driven stress response, even if corticosterone and N:L ratio are not always correlated and cannot be used interchangeably as indicators of stress (Müller et al., 2011; Davis and Maney, 2018). This could have cascading effects on multiple physiological pathways (metabolic rate, reactive oxygen species production, and immune system, Burraco and Gomez-Mestre, 2016). Consistent with this reasoning, we observed a decrease in the proportion of eosinophils with increasing salinity, which, in association with increased neutrophil and decreased lymphocyte proportions, strengthens a stress response which may be linked to increasing levels of glucocorticoid hormones (Davis and Maerz, 2010). In our study, it remains to be tested if N:L ratios are correlated with glucocorticoids hormones, as this is not always the case (Müller et al., 2011; Davis and Maney, 2018). These changes in leukocytes profiles can be the result of an increased susceptibility to infections or parasites, in correlation with other immune responses (Al-Murrani et al., 2002; Davis et al., 2008), even if the prevalence of parasites and pathogens can be reduced in saltwater (Yohannes et al., 2009; Gutiérrez, 2014; Clulow et al., 2018). However, it has to be emphasized that the increase in N:L ratios in response to infections is not consistent among amphibians species (Gervasi et al., 2014), and it remains to be tested whether it is the case in our study species. Moreover, knowledge of baseline data (not known in our study species) is critical for a thorough interpretation of leucocyte profiles (Davis and Maney, 2018). Interestingly, we showed that this response occurs rapidly (48 h), mainly in water salinity that exceeds those of sites in which we captured the study individuals. Such relatively high salinity may well explain the variable responses we observed between the field and the lab in leucocytes proportion (Table 2). It may be insightful to examine these parameters under the same range of salinity both in the field and in the lab. Because previous work has shown that salinity induces cell damage (Koleva et al., 2017), we expected (but did not find) an increase in the concentration of hemoglobin-binding proteins for saline treatments, as these proteins are expected to bind free hemoglobin and thereby reduce their detrimental consequences (Andersen et al., 2012). It is plausible that the duration of exposure to salinity in our experimental setting was short enough to spare erythrocytes (48 h compared to 84 h in Koleva et al., 2017), a hypothesis supported by the lack of damaged cells in our blood smears (pers. obs.). Hemoglobin-binding proteins have been found in some amphibians (*Taricha granulosa*, see Francis et al., 1985), but not in all (for example in *Xenopus tropicalis,* see Wicher and Fries, 2006). The role of such proteins in amphibians is still not well understood and further studies are needed in order to thoroughly describe hemoglobin-binding proteins in amphibians, and/or to develop a specific assay. Moreover, we used a kit aimed to measure binding activity to human hemoglobin, and whether frogs’ proteins are binding to the human hemoglobin remains unknown. As it is, our assay could also have measured the peroxidase activity, instead of hemoglobin-binding proteins concentration.

#### 4.2.3 Behavioural Consequences

At the end of exposure, we observed that individuals exposed to a high salt concentration expressed a decrease in activity and in jumping performance, which can have direct effects on survival, by decreasing foraging ability and anti-predator responses (Wassersug and Sperry, 1977; Squires et al., 2008; Denoël et al., 2010). Three hypotheses can explain such results. First, hyperosmolality linked to salt gain may have disrupted the processing of sensory information (Dole et al., 1994). Second, energetic costs of osmoregulation may trade-off with physical activity even if this trade-off is expected to occur over longer time-scales (i.e., several weeks, see Gomez-Mestre et al., 2004; Peña-Villalobos et al., 2016). More likely, decreased performance may be a mere consequence of dehydration (both salt gain and water loss) as this process can strongly limit locomotor abilities in amphibians (Kearney et al., 2018; Greenberg and Palen, 2021). Indeed, our results show that salt gain and performance loss are both linearly correlated with salinity. In contrast, the decreased activity scores seem to be related to water loss because both occur solely in the highest salinity tested (12 g L^−1^). Importantly, and similarly to the other parameters investigated, locomotor performance returned to initial levels when individuals are returned in freshwater, which highlights the transient effect of salinity in amphibians (Park and Do, 2020), and the high lability of their performance (Gatten and Clark, 1989).

### 4.3 Overall Salinity Impact

*Pelophylax* sp. have been described to be relatively tolerant to salinity (Natchev et al., 2011), but our data show that *Pelophylax* sp*.* adults can be affected by exposure to saline conditions. Eight individuals from the highest salinity treatment died either during treatment or shortly following their return to freshwater. This mortality was observed for smaller individuals, which suggests that a smaller body size—and thus relatively higher surface area to volume ratio—may increase susceptibility to salinity. However, we found an opposite effect of size on mass and performance changes. Indeed, at 12 g L^−1^, larger individuals lost more water and exhibited a greater decrease in their performance. During recovery, these individuals gained more mass. As smaller individuals have been shown to be more susceptible to water loss with high salinity (Gordon et al., 1961), this seems to be a counterintuitive result: due to their comparatively lower surface area to volume ratio, we would have expected larger individuals to be less susceptible to salinity. It is plausible that, for relatively high levels of salinity, the absolute surface of exchange (skin surface) may be more important to take into consideration than the surface area to volume ratio. Interestingly, although it has been shown that saline populations showed a higher osmotolerance (Licht et al., 1975; Gomez-Mestre and Tejedo, 2003), we found no effect of the salinity of the pond of origin. The lack of effect of the salinity of the pond of origin could be explained by the fact that all ponds were located on a relatively small spatial scale, and because *Pelophylax* sp. is a relatively mobile species (Wells, 2007). Such a hypothesis could be tested using continental individuals (never exposed to salinity).

## 5 Conclusion

Our study highlights the importance of considering the impact of sublethal salinity concentrations, since it could affect organismal behaviour (jumping performance and activity) and physiology (body mass, osmolality, immunology) at a very short time scale. Adult amphibians are clearly susceptible to environmental salinity, but these effects are remarkably transient once returned to freshwater. Despite the transient character of the effects that we found, we cannot rule out potential long-term effects of exposure to salinity, as it is well-known that stressful conditions, even during short time frames, can affect organismal physiology at longer time-scales (Masero et al., 2017). Further studies are required to investigate whether individuals that never experienced saline conditions (e.g., continental) could display similar short-term responses to salinity, in order to test for local adaptations to low but variable salinity. It is also critical to investigate whether exposure to environmental salinity can ultimately affect individual fitness and population persistence.

## Data Availability

The raw data supporting the conclusions of this article will be made available by the authors, without undue reservation.
