# Supplementary material for: From the Field to the Lab: Physiological and Behavioural Consequences of Environmental Salinity in a Coastal Frog
Source: Front Physiol. 2022 Jun 2;13:919165. doi: 10.3389/fphys.2022.919165 (PMC9201275; doi:10.3389/fphys.2022.919165)
Supplement: Supplementary file 1 [file DataSheet1.docx]

Appendix A: Map of the study area and global situation in Western France. The blue points represent the ponds in which frogs were captured (see Appendix B for precision on salinity and dates of capture).


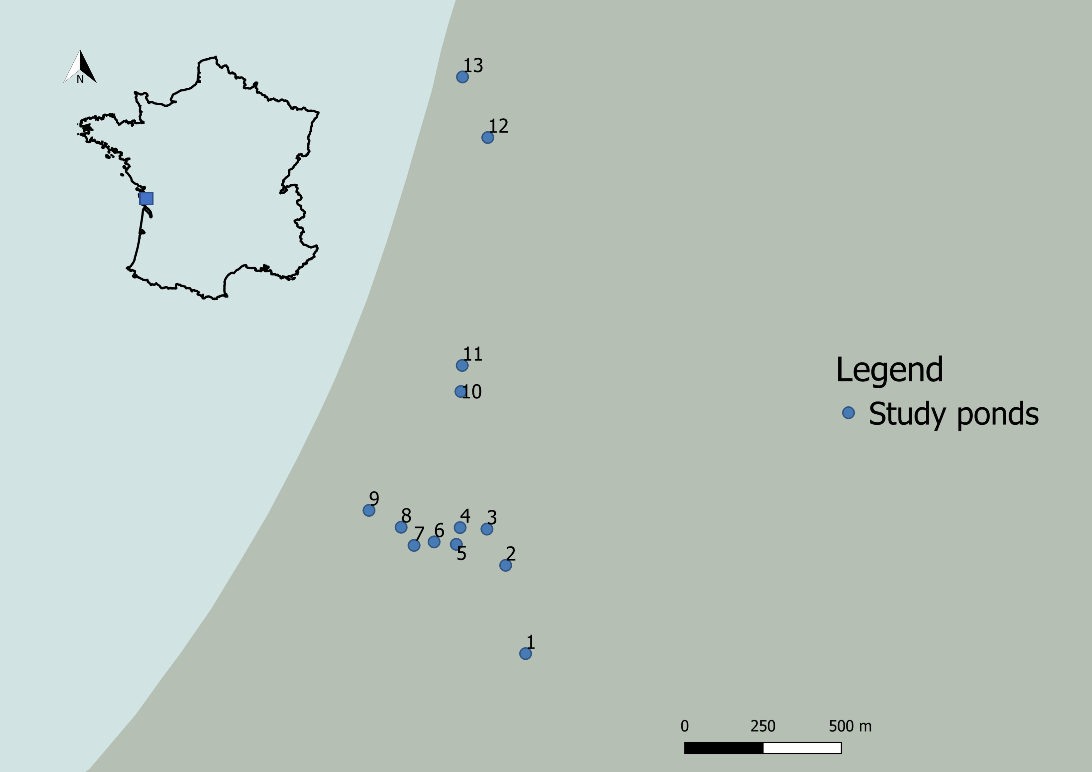


Appendix B: Pond salinity, date of capture, and number of females and males captured on each pond. See Appendix A for precise location of the ponds.

| Pond | Pond salinity  (g.l^-1^) | Date | Julian date | Females | Males | Total | Sex ratio |
| --- | --- | --- | --- | --- | --- | --- | --- |
| 1 | 0.56 | 01/05/2021 | 120 | 2 | 0 | 2 | 1 |
| 2 | 1.70 | 30/03/2021 | 88 | 1 | 0 | 1 | 1 |
| 2 | 1.90 | 19/04/2021 | 108 | 3 | 9 | 12 | 0.25 |
| 3 | 1.78 | 30/03/2021 | 88 | 2 | 0 | 2 | 1 |
| 4 | 0.10 | 01/05/2021 | 120 | 1 | 1 | 2 | 0.50 |
| 4 | 0.12 | 30/03/2021 | 88 | 2 | 1 | 3 | 0.67 |
| 5 | 0.23 | 01/05/2021 | 120 | 3 | 0 | 3 | 1 |
| 5 | 0.25 | 12/05/2021 | 131 | 3 | 1 | 4 | 0.75 |
| 6 | 2.07 | 14/04/2021 | 103 | 6 | 6 | 12 | 0.50 |
| 6 | 2.79 | 18/05/2021 | 137 | 1 | 1 | 2 | 0.50 |
| 7 | 3.89 | 18/05/2021 | 137 | 3 | 6 | 9 | 0.33 |
| 8 | 3.57 | 03/04/2021 | 92 | 3 | 7 | 10 | 0.30 |
| 8 | 4.53 | 12/05/2021 | 131 | 1 | 10 | 11 | 0.09 |
| 8 | 4.71 | 01/05/2021 | 120 | 3 | 4 | 7 | 0.43 |
| 9 | 6.53 | 23/05/2021 | 142 | 1 | 9 | 10 | 0.10 |
| 9 | 7.16 | 29/05/2021 | 148 | 6 | 10 | 16 | 0.38 |
| 10 | 0.61 | 25/04/2021 | 114 | 5 | 4 | 9 | 0.56 |
| 11 | 0.66 | 18/05/2021 | 137 | 3 | 1 | 4 | 0.75 |
| 11 | 0.73 | 25/04/2021 | 114 | 3 | 3 | 6 | 0.50 |
| 11 | 0.76 | 23/05/2021 | 142 | 1 | 2 | 3 | 0.33 |
| 12 | 3.83 | 08/04/2021 | 97 | 3 | 9 | 12 | 0.25 |
| 13 | 3.54 | 06/05/2021 | 125 | 3 | 10 | 13 | 0.23 |

Appendix C: Sample size and summary data (mean, SE, Min, Max) for size (SVL) and mass of individuals in each experimental treatment, for the whole set of individuals, or the sub-sampling of 40 individuals used for the hemoglobin-binding proteins assay and the leucocyte count.

|  |  | Total sampling | | | | | |
| --- | --- | --- | --- | --- | --- | --- | --- |
| Variable | Treatment | Mean | SE | Min | Max | N_females_ | N_males_ |
| SVL (mm) | 0 | 76,170 | 2,404 | 56 | 124 | 21 | 26 |
|  | 6 | 76,326 | 2,363 | 58 | 126 | 17 | 29 |
|  | 9 | 78,467 | 2,450 | 54 | 100 | 10 | 20 |
|  | 12 | 77,348 | 2,588 | 57 | 115 | 12 | 21 |
| Mass (g) | 0 | 48,736 | 4,652 | 15,3 | 159,8 | 21 | 26 |
|  | 6 | 46,324 | 4,858 | 16,9 | 154,5 | 17 | 29 |
|  | 9 | 45,957 | 3,919 | 15,9 | 91,1 | 10 | 20 |
|  | 12 | 47,485 | 5,477 | 14,9 | 158 | 12 | 21 |
|  |  | Sampling of 120 | | | | | |
| Variable | Treatment | Mean | SE | Min | Max | N_females_ | N_males_ |
| SVL (mm) | 0 | 80,5 | 3,097 | 56 | 124 | 5 | 5 |
|  | 6 | 83,1 | 2,996 | 58 | 126 | 4 | 6 |
|  | 9 | 83,4 | 2,919 | 56 | 100 | 3 | 7 |
|  | 12 | 80,8 | 3,236 | 57 | 115 | 5 | 5 |
| Mass (g) | 0 | 57,64 | 6,571 | 15,3 | 159,8 | 5 | 5 |
|  | 6 | 57,04 | 6,107 | 16,9 | 154,5 | 4 | 6 |
|  | 9 | 56,3 | 4,705 | 16,8 | 91,1 | 3 | 7 |
|  | 12 | 57,83 | 7,490 | 17,8 | 158 | 5 | 5 |

Appendix D: Changes of **(A)** body mass, **(B)** treatment salinity concentration, **(C)** changes in osmolality, **(D)** N:L ratio (Neutrophils/Lymphocytes ratio), and **(E)** jumping performance (relative to individuals body size: mean distance [mm]/individuals body size [mm]) of individuals during exposure.


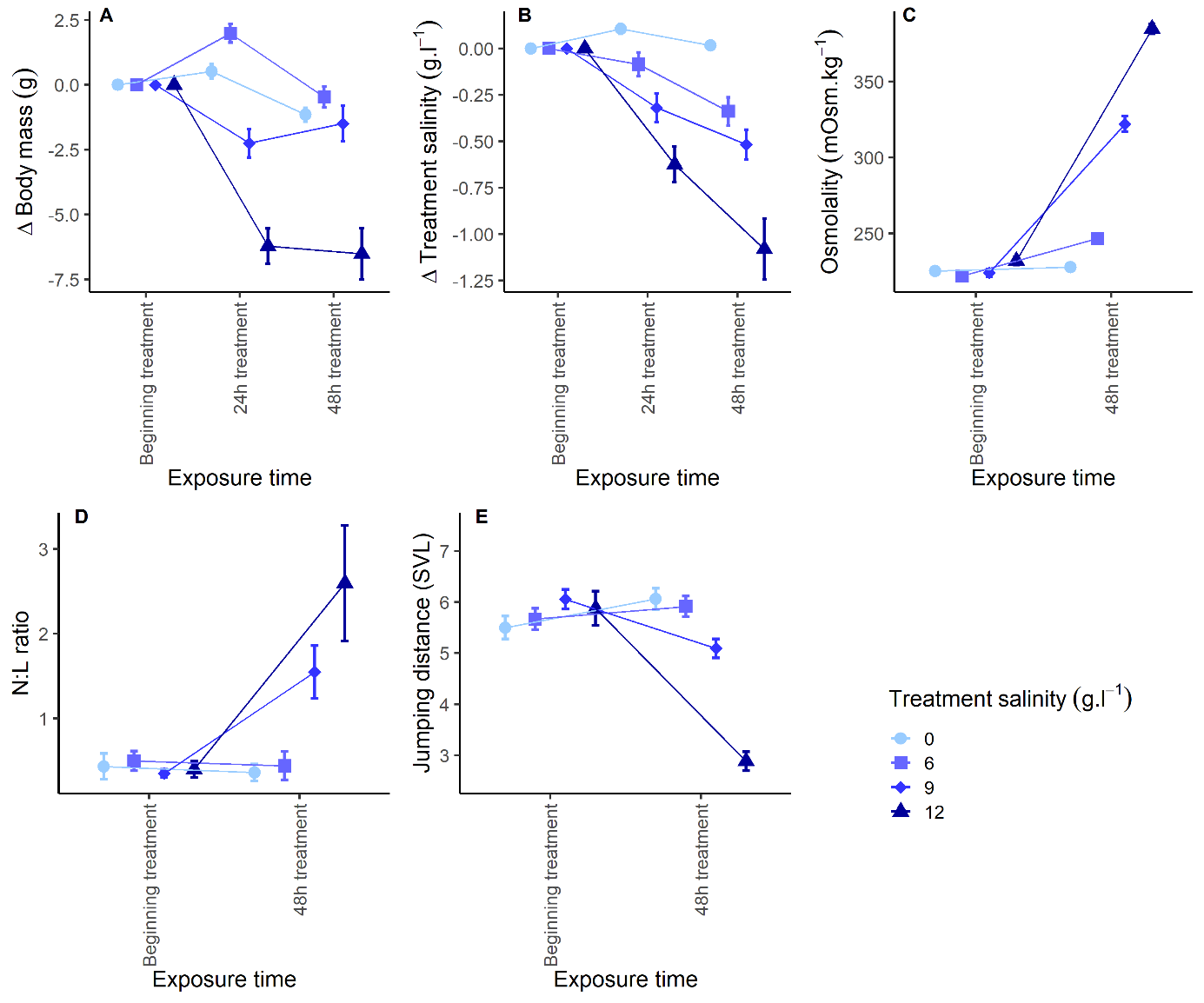


Appendix E: Changes in **(A)** body mass, **(B)** treatment salinity concentration, and **(C)** jumping performance (relative to individuals body size: mean distance [mm]/individuals body size [mm]) of individuals during recovery.


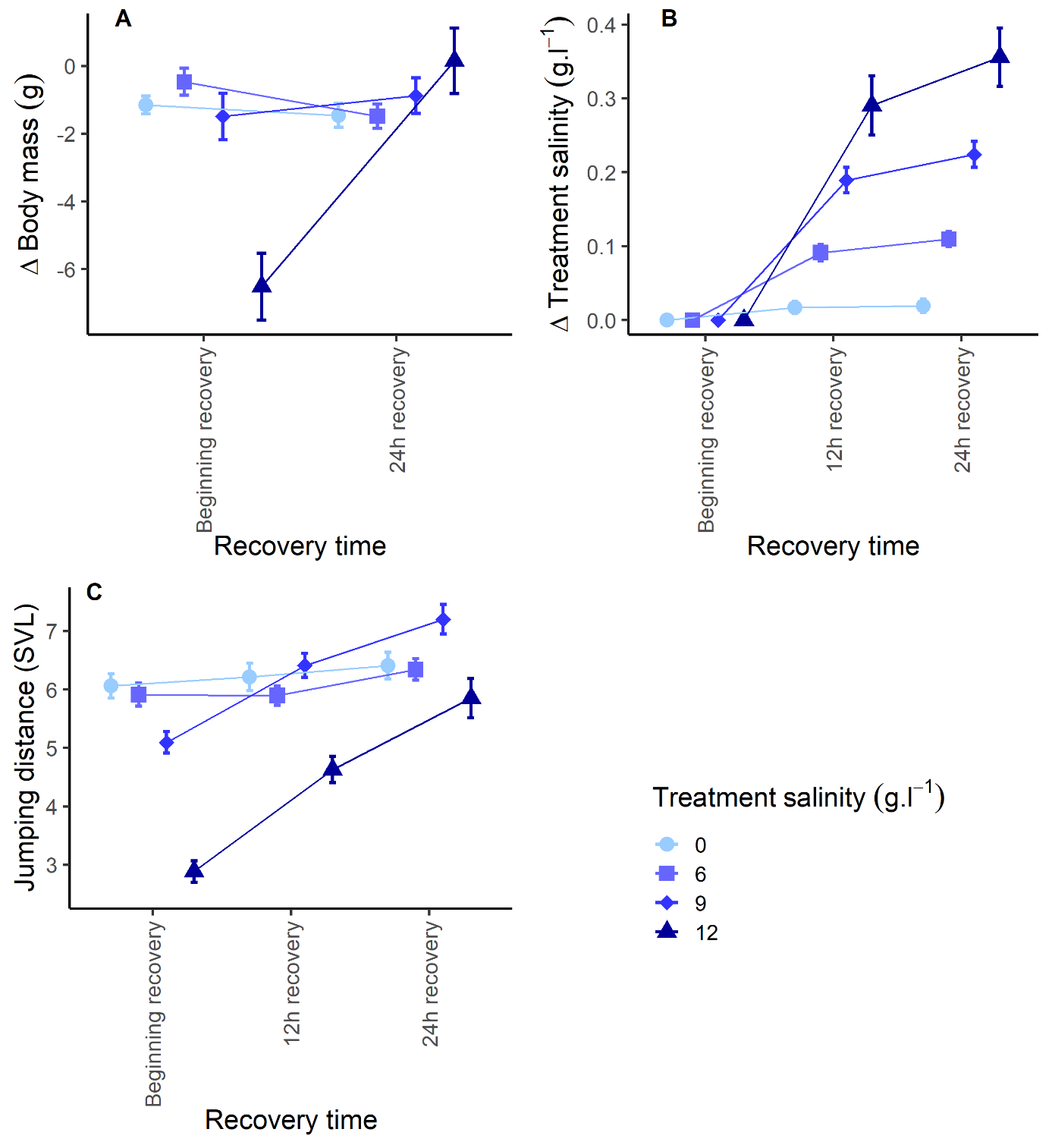


Appendix F: Effect of time in each treatment (0, 6, 9 and 12 g.l^-1^ salinity treatments) on body mass changes, osmolality, changes in treatment concentration, N:L ratio, in the different leucocytes proportion, jumping performance and activity during exposure, , computed on N=156 individuals (0 g.l^-1^: N=47; 6 g.l^-1^: N=46; 9 g.l^-1^: N=30; 12 g.l^-1^: N=33), except for hemoglobin-binding proteins and leucocytes counts, where it was computed on N=40 individuals (10 per treatments).

|  | time*treatment | | |  |  | Dunnett post hoc test | | | |
| --- | --- | --- | --- | --- | --- | --- | --- | --- | --- |
| Comparison | F-value | Chisq | p-value | Treatment | Time | Estimate | SE | t/z | p-value |
| Body mass changes | 31.156 | - | <0.001 | 0 | Beginning exposure-24 hours exposure | -0.519 | 0.430 | -1.207 | 0.449 |
|  |  |  |  |  | Beginning exposure-48 hours exposure | 1.151 | 0.430 | 2.677 | 0.021 |
|  |  |  |  |  | 24 hours exposure- 48 hours exposure | 1.670 | 0.430 | 3.884 | <0.001 |
|  |  |  |  | 6 | Beginning exposure-24 hours exposure | -1.989 | 0.435 | -4.576 | <0.001 |
|  |  |  |  |  | Beginning exposure-48 hours exposure | 0.470 | 0.435 | 1.080 | 0.527 |
|  |  |  |  |  | 24 hours exposure- 48 hours exposure | 2.459 | 0.435 | 5.656 | <0.001 |
|  |  |  |  | 9 | Beginning exposure-24 hours exposure | 2.250 | 0.538 | 4.180 | <0.001 |
|  |  |  |  |  | Beginning exposure-48 hours exposure | 1.490 | 0.538 | 2.768 | 0.016 |
|  |  |  |  |  | 24 hours exposure- 48 hours exposure | -0.76 | 0.538 | -1.412 | 0.336 |
|  |  |  |  | 12 | Beginning exposure-24 hours exposure | 6.221 | 0.513 | 12.122 | <0.001 |
|  |  |  |  |  | Beginning exposure-48 hours exposure | 6.270 | 0.537 | 11.666 | <0.001 |
|  |  |  |  |  | 24 hours exposure- 48 hours exposure | 0.049 | 0.537 | 0.090 | 0.996 |
| Treatment concentration | 14.429 | - | <0.001 | 0 | Beginning exposure-24 hours exposure | -0.106 | 0.078 | -1.36 | 0.364 |
|  |  |  |  |  | Beginning exposure-48 hours exposure | -0.017 | 0.078 | -0.224 | 0.973 |
|  |  |  |  |  | 24 hours exposure- 48 hours exposure | 0.088 | 0.078 | 1.136 | 0.493 |
|  |  |  |  | 6 | Beginning exposure-24 hours exposure | 0.086 | 0.079 | 1.089 | 0.522 |
|  |  |  |  |  | Beginning exposure-48 hours exposure | 0.338 | 0.079 | 4.274 | <0.001 |
|  |  |  |  |  | 24 hours exposure- 48 hours exposure | 0.253 | 0.079 | 3.192 | 0.004 |
|  |  |  |  | 9 | Beginning exposure-24 hours exposure | 0.319 | 0.097 | 3.272 | 0.003 |
|  |  |  |  |  | Beginning exposure-48 hours exposure | 0.518 | 0.097 | 5.317 | <0.001 |
|  |  |  |  |  | 24 hours exposure- 48 hours exposure | 0.199 | 0.097 | 2.045 | 0.104 |
|  |  |  |  | 12 | Beginning exposure-24 hours exposure | 0.625 | 0.093 | 6.727 | <0.001 |
|  |  |  |  |  | Beginning exposure-48 hours exposure | 1.069 | 0.098 | 10.936 | <0.001 |
|  |  |  |  |  | 24 hours exposure- 48 hours exposure | 0.443 | 0.098 | 4.541 | <0.001 |
| Osmolality | 305.12 | - | <0.001 | 0 | Beginning exposure-48 hours exposure | -2.71 | 3.560 | -0.739 | 0.727 |
|  |  |  |  | 6 | Beginning exposure-48 hours exposure | -24.778 | 3.520 | -7.114 | <0.001 |
|  |  |  |  | 9 | Beginning exposure-48 hours exposure | -98.07 | 4.250 | -23.35 | <0.001 |
|  |  |  |  | 12 | Beginning exposure-48 hours exposure | -153.96 | 4.390 | -35.372 | <0.001 |
| Lymphocytes proportion | - | 86.839 | <0.001 | 0 | Beginning exposure-48 hours exposure | -0.015 | 0.077 | -0.199 | 0.842 |
|  |  |  |  | 6 | Beginning exposure-48 hours exposure | -0.162 | 0.076 | -2.14 | 0.032 |
|  |  |  |  | 9 | Beginning exposure-48 hours exposure | 0.488 | 0.079 | 6.166 | <0.001 |
|  |  |  |  | 12 | Beginning exposure-48 hours exposure | 0.775 | 0.087 | 8.895 | <0.001 |
| Neutrophils proportion | - | 91.745 | <0.001 | 0 | Beginning exposure-48 hours exposure | 0.053 | 0.121 | 0.438 | 0.662 |
|  |  |  |  | 6 | Beginning exposure-48 hours exposure | 0.136 | 0.110 | 1.232 | 0.218 |
|  |  |  |  | 9 | Beginning exposure-48 hours exposure | -0.838 | 0.095 | -8.825 | <0.001 |
|  |  |  |  | 12 | Beginning exposure-48 hours exposure | -0.969 | 0.094 | -10.312 | <0.001 |
| N:L ratio | 7.589 | - | <0.001 | 0 | Beginning exposure-48 hours exposure | 0.071 | 0.397 | 0.179 | 0.859 |
|  |  |  |  | 6 | Beginning exposure-48 hours exposure | 0.058 | 0.397 | 0.145 | 0.886 |
|  |  |  |  | 9 | Beginning exposure-48 hours exposure | -1.197 | 0.397 | -3.012 | 0.005 |
|  |  |  |  | 12 | Beginning exposure-48 hours exposure | -2.195 | 0.397 | -5.522 | <0.001 |
| Eosinophils proportion | - | 18.601 | <0.001 | 0 | Beginning exposure-48 hours exposure | 0.198 | 0.163 | 1.216 | 0.224 |
|  |  |  |  | 6 | Beginning exposure-48 hours exposure | -0.107 | 0.188 | -0.572 | 0.567 |
|  |  |  |  | 9 | Beginning exposure-48 hours exposure | 0.858 | 0.247 | 3.47 | <0.001 |
|  |  |  |  | 12 | Beginning exposure-48 hours exposure | 1.101 | 0.273 | 4.035 | <0.001 |
| Monocytes proportion | - | 3.552 | 0.314 | - | - | - | - | - | - |
| Basophils proportion | - | 1.698 | 0.637 | - | - | - | - | - | - |
| Hemoglobin-binding protein concentration | 0.768 | - | 0.52 | - | - | - | - | - | - |
| Jumping performance | 70.85 | - | <0.001 | 0 | Beginning exposure-48 hours exposure | -0.561 | 0.158 | -3.547 | <0.001 |
|  |  |  |  | 6 | Beginning exposure-48 hours exposure | -0.246 | 0.160 | -1.539 | 0.126 |
|  |  |  |  | 9 | Beginning exposure-48 hours exposure | 0.958 | 0.198 | 4.838 | <0.001 |
|  |  |  |  | 12 | Beginning exposure-48 hours exposure | 2.901 | 0.203 | 14.313 | <0.001 |
| Activity | - | 18.279 | <0.001 | 0 | Beginning exposure-48 hours exposure | 0.007 | 0.134 | 0.056 | 0.956 |
|  |  |  |  | 6 | Beginning exposure-48 hours exposure | <0.001 | 0.133 | -0.003 | 0.998 |
|  |  |  |  | 9 | Beginning exposure-48 hours exposure | 0.034 | 0.164 | 0.207 | 0.836 |
|  |  |  |  | 12 | Beginning exposure-48 hours exposure | 0.860 | 0.181 | -4.75 | <0.001 |

Appendix G: Effect of time in each treatment (0, 6, 9 and 12 g.l^-1^ salinity treatments) on body mass changes, changes in recovery treatment concentration, jumping performance and activity during recovery, , computed on N=156 individuals (0 g.l^-1^: N=47; 6 g.l^-1^: N=46; 9 g.l^-1^: N=30; 12 g.l^-1^: N=33), except for hemoglobin-binding proteins and leucocytes counts, where it was computed on N=40 individuals (10 per treatments).

|  | time*treatment | | |  |  | Dunnett post hoc test | | | |
| --- | --- | --- | --- | --- | --- | --- | --- | --- | --- |
| Comparison | F-value | Chisq | p-value | Treatment | Time | Estimate | SE | t/z | p-value |
| Body mass changes | 6.777 | - | <0.001 | 0 | Beginning exposure- End of recovery | 1.404 | 0.465 | 3.018 | 0.003 |
|  |  |  |  |  | 48 hours exposure- End of recovery | 0.222 | 0.441 | 0.502 | 0.616 |
|  |  |  |  | 6 | Beginning exposure- End of recovery | 1.469 | 0.457 | 3.218 | 0.002 |
|  |  |  |  |  | 48 hours exposure- End of recovery | 0.947 | 0.432 | 2.191 | 0.03 |
|  |  |  |  | 9 | Beginning exposure- End of recovery | 0.877 | 0.55 | 1.594 | 0.113 |
|  |  |  |  |  | 48 hours exposure- End of recovery | -0.613 | 0.52 | -1.18 | 0.24 |
|  |  |  |  | 12 | Beginning exposure- End of recovery | -0.202 | 0.589 | -0.344 | 0.732 |
|  |  |  |  |  | 48 hours exposure- End of recovery | -6.661 | 0.564 | -11.812 | <0.001 |
| Recovery concentration | 28.651 | - | <0.001 | 0 | 48 hours exposure- 12 hours recovery | -0.017 | 0.014 | -1.185 | 0.463 |
|  |  |  |  |  | 48 hours exposure- End of recovery | -0.019 | 0.015 | -1.311 | 0.39 |
|  |  |  |  |  | 12 hours recovery- End of recovery | -0.002 | 0.015 | -0.134 | 0.99 |
|  |  |  |  | 6 | 48 hours exposure- 12 hours recovery | -0.091 | 0.014 | -6.431 | <0.001 |
|  |  |  |  |  | 48 hours exposure- End of recovery | -0.11 | 0.014 | -7.725 | <0.001 |
|  |  |  |  |  | 12 hours recovery- End of recovery | -0.018 | 0.014 | -1.294 | 0.4 |
|  |  |  |  | 9 | 48 hours exposure- 12 hours recovery | -0.189 | 0.017 | -11.144 | <0.001 |
|  |  |  |  |  | 48 hours exposure- End of recovery | -0.224 | 0.017 | -13.183 | <0.001 |
|  |  |  |  |  | 12 hours recovery- End of recovery | -0.035 | 0.017 | -2.039 | 0.105 |
|  |  |  |  | 12 | 48 hours exposure- 12 hours recovery | -0.288 | 0.019 | -15.587 | <0.001 |
|  |  |  |  |  | 48 hours exposure- End of recovery | -0.35 | 0.019 | -18.75 | <0.001 |
|  |  |  |  |  | 12 hours recovery- End of recovery | -0.062 | 0.019 | -3.317 | 0.003 |
| Jumping performance | 38.178 | - | <0.001 |  | Beginning exposure- 12 hours recovery | -0.546 | 0.152 | -3.605 | 0.001 |
|  |  |  |  | 0 | Beginning exposure- End of recovery | -0.737 | 0.152 | -4.864 | <0.001 |
|  |  |  |  |  | 48 hours exposure- 12 hours recovery | -0.06 | 0.138 | -0.435 | 0.901 |
|  |  |  |  |  | 48 hours exposure- End of recovery | -0.251 | 0.138 | -1.822 | 0.614 |
|  |  |  |  |  | 12 hours recovery- End of recovery | -0.191 | 0.139 | -1.377 | 0.354 |
|  |  |  |  | 6 | Beginning exposure- 12 hours recovery | -0.105 | 0.148 | -0.708 | 0.759 |
|  |  |  |  |  | Beginning exposure- End of recovery | -0.549 | 0.148 | -3.704 | <0.001 |
|  |  |  |  |  | 48 hours exposure- 12 hours recovery | 0.114 | 0.135 | 0.847 | 0.674 |
|  |  |  |  |  | 48 hours exposure- End of recovery | -0.331 | 0.135 | -2.453 | 0.039 |
|  |  |  |  |  | 12 hours recovery- End of recovery | -0.445 | 0.135 | -3.288 | 0.003 |
|  |  |  |  | 9 | Beginning exposure- 12 hours recovery | -0.36 | 0.178 | -2.017 | 0.11 |
|  |  |  |  |  | Beginning exposure- End of recovery | -1.15 | 0.178 | -6.446 | <0.001 |
|  |  |  |  |  | 48 hours exposure- 12 hours recovery | -1.318 | 0.162 | -8.136 | <0.001 |
|  |  |  |  |  | 48 hours exposure- End of recovery | -2.108 | 0.162 | -13.015 | <0.001 |
|  |  |  |  |  | 12 hours recovery- End of recovery | -0.79 | 0.162 | -4.879 | <0.001 |
|  |  |  |  | 12 | Beginning exposure- 12 hours recovery | 1.211 | 0.2 | 6.045 | <0.001 |
|  |  |  |  |  | Beginning exposure- End of recovery | -0.011 | 0.2 | -0.052 | 0.999 |
|  |  |  |  |  | 48 hours exposure- 12 hours recovery | -1.771 | 0.183 | -9.671 | <0.001 |
|  |  |  |  |  | 48 hours exposure- End of recovery | -2.993 | 0.183 | -16.339 | <0.001 |
|  |  |  |  |  | 12 hours recovery- End of recovery | -1.221 | 0.185 | -6.602 | <0.001 |
| Activity | - | 15.683 | 0.016 | 0 | Beginning exposure- 12 hours recovery | -0.014 | 0.137 | -0.102 | 0.994 |
|  |  |  |  |  | Beginning exposure- End of recovery | -0.009 | 0.138 | -0.066 | 0.998 |
|  |  |  |  |  | 48 hours exposure- 12 hours recovery | -0.022 | 0.135 | -0.16 | 0.986 |
|  |  |  |  |  | 48 hours exposure- End of recovery | -0.017 | 0.136 | -0.123 | 0.992 |
|  |  |  |  |  | 12 hours recovery- End of recovery | 0.005 | 0.139 | 0.035 | 0.999 |
|  |  |  |  | 6 | Beginning exposure- 12 hours recovery | 0.006 | 0.136 | 0.041 | 0.999 |
|  |  |  |  |  | Beginning exposure- End of recovery | -0.018 | 0.135 | -0.133 | 0.99 |
|  |  |  |  |  | 48 hours exposure- 12 hours recovery | 0.006 | 0.135 | 0.004 | 0.999 |
|  |  |  |  |  | 48 hours exposure- End of recovery | -0.018 | 0.134 | -0.13 | 0.991 |
|  |  |  |  |  | 12 hours recovery- End of recovery | -0.024 | 0.137 | -0.172 | 0.984 |
|  |  |  |  | 9 | Beginning exposure- 12 hours recovery | 0.007 | 0.163 | 0.041 | 0.999 |
|  |  |  |  |  | Beginning exposure- End of recovery | <0.001 | 0.163 | <0.001 | 0.999 |
|  |  |  |  |  | 48 hours exposure- 12 hours recovery | -0.027 | 0.164 | -0.166 | 0.985 |
|  |  |  |  |  | 48 hours exposure- End of recovery | -0.034 | 0.164 | -0.207 | 0.977 |
|  |  |  |  |  | 12 hours recovery- End of recovery | -0.007 | 0.163 | -0.041 | 0.999 |
|  |  |  |  | 12 | Beginning exposure- 12 hours recovery | 0.221 | 0.172 | 1.282 | 0.405 |
|  |  |  |  |  | Beginning exposure- End of recovery | 0.032 | 0.173 | 0.186 | 0.981 |
|  |  |  |  |  | 48 hours exposure- 12 hours recovery | -0.639 | 0.195 | -3.28 | 0.003 |
|  |  |  |  |  | 48 hours exposure- End of recovery | -0.827 | 0.196 | -4.226 | <0.001 |
|  |  |  |  |  | 12 hours recovery- End of recovery | -0.188 | 0.188 | -1.004 | 0.574 |

Appendix H: Proportion of the different leucocytes for wild frogs, computed on N=40 individuals.

| Proportion of the different leucocytes | Mean | SE |
| --- | --- | --- |
| N:L ratio | 0.252 | 0.028 |
| % Lymphocytes | 59.525 | 2.117 |
| % Neutrophils | 13.575 | 1.137 |
| % Monocytes | 16.425 | 1.582 |
| % Eosinophiles | 9.95 | 10.667 |
| % Basophiles | 0.525 | 0.156 |

Appendix I: Changes in body mass, osmolality, N:L ratio, leucocytes proportion, hemoglobin-binding protein concentration, jumping performance and activity, during acclimation, and comparisons between the different salinities at the end of acclimation. We also tested that body size (SVL) and sex were equivalent between exposures. Computed on N=156 individuals (0 g.l^-1^: N=47; 6 g.l^-1^: N=46; 9 g.l^-1^: N=30; 12 g.l^-1^: N=33), except for hemoglobin-binding proteins and leucocytes counts, where it was computed on N=40 individuals (10 per treatments).

|  | Variations during acclimation | | | | Comparison between exposures | | | | |
| --- | --- | --- | --- | --- | --- | --- | --- | --- | --- |
| Comparison | Estimate | SE | t/z | p-value | Df | Sum Sq | F-value | Chisq | p-value |
| SVL | - | - | - | - | 3 | 36564 | 0.167 | - | 0.919 |
| Sex | - | - | - | - | 3 | - | - | 0.397 | 0.941 |
| Body mass | -3.95 | 0.337 | -11.73 | <0.001 | 3 | 39 | 0.018 | - | 0.997 |
| Osmolality | -4.185 | 1.76 | -2.378 | 0.0174 | 3 | 1937 | 2.798 | - | 0.042 |
| Lymphocytes proportion | -0.069 | 0.037 | -1.873 | 0.061 | 3 | - | - | 3.925 | 0.269 |
| Neutrophils proportion | 0.374 | 0.06 | 6.207 | <0.001 | 3 | - | - | 2.093 | 0.553 |
| N:L ratio | 0.169 | 0.059 | 2.863 | 0.004 | 3 | 0.116 | 0.321 | - | 0.81 |
| Eosinophils proportion | -0.406 | 0.084 | -4.822 | <0.001 | 3 | - | - | 1.846 | 0.605 |
| Monocytes proportion | 0.023 | 0.06 | 0.382 | 0.703 | 3 | - | - | 7.302 | 0.063 |
| Basophils proportion | -0.478 | 0.354 | -1.349 | 0.177 | 3 | - | - | 1.354 | 0.716 |
| Hemoglobin-binding protein concentration | 0.059 | 0.031 | 1.922 | 0.055 | 3 | 0.15 | 1.07 | - | 0.374 |
| Jumping performance | 0.439 | 0.067 | 6.596 | <0.001 | 3 | 6.5 | 0.945 | - | 0.421 |
| Activity | 0.021 | 0.074 | 0.29 | 0.772 | 3 | 0.149 | 0.882 | - | 0.452 |

Appendix J: Effect of time, treatment, size and the interaction between time and treatment, and between treatment and size, on body mass changes, osmolality, changes in treatment concentration (during exposure and recovery), N:L ratio, leucocytes counts, jumping performance and activity. Note that only the covariates that are retained in the backward selection are retained (pond salinity and sex of individuals were set in the global models, but not retained during model selection procedure). Computed on N=156 individuals (0 g.l^-1^: N=47; 6 g.l^-1^: N=46; 9 g.l^-1^: N=30; 12 g.l^-1^: N=33).

| Comparison | Time | Covariate | SumSq | Df | F-value | Chisq | p-value |
| --- | --- | --- | --- | --- | --- | --- | --- |
| Body mass changes | Exposure | Time | 425.91 | 1 | 74.738 | - | <0.001 |
|  |  | Treatment | 54.92 | 3 | 3.212 | - | 0.025 |
|  |  | Time*Treatment | 383.39 | 3 | 22.425 | - | <0.001 |
|  | Recovery | Time | 155.920 | 1 | 38.447 | - | <0.001 |
|  |  | Treatment | 69.840 | 3 | 5.741 | - | <0.001 |
|  |  | Size | 942.960 | 1 | 232.517 | - | <0.001 |
|  |  | Time*Treatment | 524.810 | 3 | 43.136 | - | <0.001 |
|  |  | Size*Treatment | 82.450 | 3 | 6.777 | - | <0.001 |
| Treatment concentration | Exposure | Time | 18.824 | 1 | 130.229 | - | <0.001 |
|  |  | Treatment | 11.405 | 3 | 29.444 | - | <0.001 |
|  |  | Time*Treatment | 11.405 | 3 | 29.444 | - | <0.001 |
|  | Recovery | Time | 2.056 | 1 | 506.767 | - | <0.001 |
|  |  | Treatment | 0.061 | 3 | 4.999 | - | 0.002 |
|  |  | Size | 0.292 | 1 | 71.945 | - | <0.001 |
|  |  | Time*Treatment | 0.979 | 3 | 80.426 | - | <0.001 |
|  |  | Size*Treatment | 0.19 | 3 | 15.619 | - | <0.001 |
| Osmolality | Exposure | Time | 337875 | 1 | 1276.97 | - | <0.001 |
|  |  | Treatment | 213814 | 3 | 269.36 | - | <0.001 |
|  |  | Time*Treatment | 242195 | 3 | 305.12 | - | <0.001 |
| Lymphocytes proportion | Exposure | Time | - | 1 | - | 34.920 | <0.001 |
|  |  | Treatment | - | 3 | - | 8.178 | 0.042 |
|  |  | Time*Treatment | - | 3 | - | 86.839 | <0.001 |
| Neutrophils proportion | Exposure | Time | - | 1 | - | 94.181 | <0.001 |
|  |  | Treatment | - | 3 | - | 43.587 | <0.001 |
|  |  | Time*Treatment | - | 3 | - | 91.745 | <0.001 |
| N:L ratio | Exposure | Time | 13.305 | 1 | 16.848 | - | <0.001 |
|  |  | Treatment | 14.776 | 3 | 6.237 | - | 0.002 |
|  |  | Time*Treatment | 17.978 | 3 | 7.589 | - | <0.001 |
| Eosinophils proportion | Exposure | Time | - | 1 | - | 11.524 | <0.001 |
|  |  | Treatment | - | 3 | - | 4.528 | 0.210 |
|  |  | Time*Treatment | - | 3 | - | 18.601 | <0.001 |
| Monocytes proportion | Exposure | Time | - | 1 | - | 0.378 | 0.539 |
|  |  | Treatment | - | 3 | - | 9.226 | 0.026 |
|  |  | Time*Treatment | - | 3 | - | 3.552 | 0.314 |
| Basophils proportion | Exposure | Time | - | 1 | - | 0.124 | 0.725 |
|  |  | Treatment | - | 3 | - | 3.533 | 0.317 |
|  |  | Time*Treatment | - | 3 | - | 1.698 | 0.637 |
| Hemoglobin-binding protein concentration | Exposure | Time | 0.001 | 1 | 0.034 | - | 0.854 |
|  |  | Treatment | 0.003 | 3 | 0.103 | - | 0.751 |
|  |  | Time*Treatment | 0.001 | 3 | 0.028 | - | 0.869 |
| Jumping performance | Exposure | Time | 41.109 | 1 | 70.163 | - | <0.001 |
|  |  | Treatment | 22.619 | 3 | 7.54 | - | <0.001 |
|  |  | Size | 50.863 | 1 | 86.812 | - | <0.001 |
|  |  | Time*Treatment | 123.592 | 3 | 70.314 | - | <0.001 |
|  | Recovery | Time | 137.798 | 1 | 274.995 | - | <0.001 |
|  |  | Treatment | 32.412 | 3 | 21.561 | - | <0.001 |
|  |  | Size | 49.153 | 1 | 98.092 | - | <0.001 |
|  |  | Time*Treatment | 82.578 | 3 | 54.932 | - | <0.001 |
| Activity | Exposure | Time | - | 1 | - | 4.332 | 0.037 |
|  |  | Treatment | - | 3 | - | 11.966 | 0.008 |
|  |  | Time*Treatment | - | 3 | - | 18.279 | <0.001 |
|  | Recovery | Time | - | 1 | - | 3.531 | 0.06 |
|  |  | Treatment | - | 3 | - | 15.918 | 0.001 |
|  |  | Time*Treatment | - | 3 | - | 14.399 | 0.002 |
